# Different Crystalline Populations for Biopolyesters within Graphene-Based Nanopapers

**DOI:** 10.1021/acs.macromol.5c03526

**Published:** 2026-03-02

**Authors:** Hui Zhao, Ricardo A. Pérez-Camargo, Yongzheng Li, Zhibo Li, Guoming Liu, Alejandro J. Müller, Alberto Fina

**Affiliations:** † Dipartimento di Scienza Applicata e Tecnologia, 19032 Politecnico di Torino- Alessandria campus, viale Teresa Michel, 5, 15121 Alessandria, Italy; ‡ POLYMAT and Department of Polymers and Advanced Materials: Physics, Chemistry and Technology, Faculty of Chemistry, University of the Basque Country UPV/EHU, Paseo Manuel de Lardizabal 3, 20018, Donostia-San Sebastián, Spain; § State Key Laboratory of Advanced Optical Polymer and Manufacturing Technology; College of Polymer Science and Engineering, 66280 Qingdao University of Science and Technology, 266042, Qingdao, China; ∥ CAS Key Laboratory of Engineering Plastics, Beijing National Laboratory for Molecular Sciences, 53030 Institute of Chemistry, Chinese Academy of Sciences, Beijing 100190, China; ⊥ University of Chinese Academy of Sciences, Beijing 100049, China; # IKERBASQUE, Basque Foundation for Science, Plaza Euskadi 5, Bilbao, 48009, Spain

## Abstract

The control and design of the semicrystalline structure of polymer binders within nanopapers based on graphene-related materials (GRM) may have a significant impact on the nanopapers’ physical properties, including thermomechanical resistance and thermal conductivity. In this article, biopolyesters differing in methylene chain length between ester groups were studied, specifically using poly­(ε-caprolactone) (PCL) and poly-4-hydroxybutyrate (P4HB), with additional comparisons to polyglycolide (PGA). The crystallization behavior and crystalline structure of the polymers embedded in GRM nanopapers were studied by differential scanning calorimetry (DSC) and wide-angle X-ray scattering (WAXS). In particular, high melting point crystals originating from strong nucleation and strong molecular interactions with the GRM were observed with thermal stability dependent on the chemical structure of the polymer. The crystals having the highest melting temperatures, well above the equilibrium melting points of PCL and P4HB, are of particular interest. Besides their high thermal stability, these crystals cannot be fractionated through successive self-nucleation and annealing. At the same time, WAXS revealed distinct crystal diffraction reflections and relatively broad rings, suggesting the formation of crystals stabilized up to high temperatures by their interfacial adsorption onto GRM. These findings offer new insights into the mechanism of polymer crystallization at the interface with nanoparticles and may have implications for the development and application of hybrid organic/inorganic flexible nanopapers in electronic devices.

## Graphical abstract

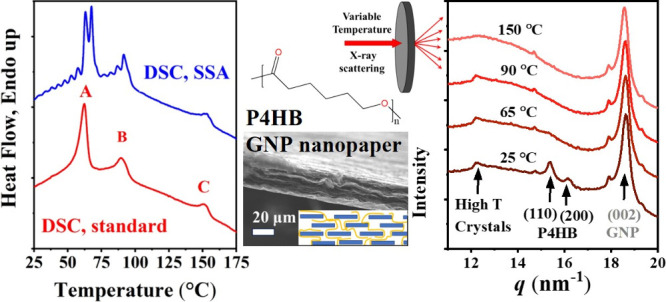

## 1 Introduction

Recent studies have shown that graphene-related materials (GRM) exhibit diverse nucleation and crystallization behavior when incorporated into polymers.^1–7^ With large specific interfacial areas, GRM are typically very effective in enhancing the crystallization temperature and crystallization rate of polymers.^8,9^ In some cases, GRM are more efficient at nucleating a polymer than their own self-nuclei, a condition referred to as supernucleation.^2,10–13^ For example, reduced graphene oxide (rGO) added to poly­(butylene terephthalate) (pCBT) significantly increased the nucleation efficiency up to 270%,^2,10^ indicating that rGO flakes are much more efficient in nucleating pCBT in comparison to the polymer self-nuclei. In addition to conventional nucleation, it was reported^14^ that, while polymer is still above its melting point, an ordered crystalline thin layer may be formed on the surface of selected solid substrates, which has been referred to as prefreezing. Formation of such a thin crystalline layer at the interface between the polymer melt and the substrate was thermodynamically justified in terms of interfacial energy balance between polymer melt/polymer crystals, polymer crystal/substrate and polymer melt/substrate.^5,15,16^ It is particularly relevant to the present work that prefreezing was reported for poly­(ε-caprolactone) (PCL) on highly oriented pyrolytic graphite (HOPG).^15,17^ As the temperature increases, this interfacial crystalline layer disappears at a well-defined melting temperature (ca. 82 °C for PCL on HOPG, about 20 °C higher than bulk PCL) and reversibly reappears upon cooling, resulting in a highly oriented and uniform crystalline structure throughout the substrate surface. This indicates that crystallization of polymers in the presence of solid particles may not only occur via conventional homogeneous and heterogeneous nucleation mechanisms but may also depend on specific interactions at the interface. These may be particularly important when polymer chains are in close contact with high surface area 2D nanoparticles.

In the case of GRM nanopapers, where a limited number of polymer chains are embedded as a binder between nanoplates, because of the high volume ratio between GRM and polymer, virtually all polymer chains are in close proximity to the surface of the inorganic particles. This can affect the conformation and mobility of the chains, in turn modifying the crystallization kinetics and structure.^18^ Li et al.^3^ first reported multiple crystalline populations obtained for PCL within graphite nanoplates (GNP) nanopapers, as evidenced by four different signals in DSC. These were assigned to conventional unoriented crystals, oriented crystals and a prefrozen crystalline layer onto the GRM surface, in agreement with evidence reported for PCL crystallization onto HOPG and other 2D substrates.^5,14,15,17^ Remarkably, a fourth signal in DSC plots was observed at a temperature significantly higher than the equilibrium melting temperature of PCL. This was tentatively explained assuming the formation of a prefrozen layer within the galleries between GNP flakes or extended chain crystals, based on a strong adsorption of PCL onto GNP.^3^ Recently, we reported the influence of PCL molecular weight, GRM type, and preparation method on the crystallization behavior.^4^ This allowed confirmation of the presence of the four crystalline populations for PCL within GRM nanopapers. It was also found that the relative intensities to the fourth peak were influenced by the defectiveness of the GRM surface and the strength of the interfacial interactions.

The objective of this work is to elucidate the impact of the chemical structure of polymers on the crystallization behavior of the composites. Three polymers are compared, including PCL, poly­(4-hydroxybutyrate) (P4HB), and polyglycolide (PGA), which differ in the number of methylene units in their repeating units, to vary the polymer/GRM interactions. First, techniques such as DSC and in situ WAXS were utilized to compare the temperature dependence of crystal structures formed by PCL, P4HB, and PGA on graphene nanosheets. Additionally, successive self-nucleation and annealing (SSA) and variable-temperature WAXS were performed to evaluate the thermal fractionation capacity of the crystals formed and their possible orientation. This study allowed to generalize to different polymers the occurrence of crystalline fractions stable up to temperatures well above the equilibrium melting temperatures. These results offer new insights into polymer structure control and material design based on polymer crystal engineering.

## 2 Experimental Section

### 2.1 Materials

Poly­(ε-caprolactone) (PCL), with a number-average molecular weight of *M*_n_ = 50000 g/mol (commercial grade CAPA 6500), was purchased from Ingevity. Poly­(4-hydroxybutyrate) (P4HB) was specially synthesized for this work according to a previously reported procedure.^19^ The final polymer produced had a number-average molecular weight (*M*_n_) of 68300 g/mol and a dispersity (*Đ*) of 1.98, determined by SEC. Polyglycolide (PGA) with an inherent viscosity of 1.4 dL/g was purchased from Sigma. The graphite nanoplates (GNP) and reduced graphene oxide (rGO) flakes used in this study were provided by Avanzare Innovacion Tecnologica S.L. (Navarrete, La Rioja, Spain) and synthesized following previously reported procedures. Briefly, GNP was produced through rapid thermal expansion of overoxidized, intercalated graphite, while rGO was obtained by oxidizing natural graphite, followed by tip-sonication in an aqueous solution and subsequent thermal reduction at 1060 °C in an argon atmosphere. Comprehensive characterization of both materials has been previously reported.^20,21^ Dimethylformamide (DMF, ≥99.8%, Merck or Carlo Erba Reagents) and hexafluoroisopropanol (HFIP, ≥99.5%, Merck) were used as received.

### 2.2 Preparation Methods

#### 2.2.1 Preparation of Nanopapers by Filtration

The preparation procedures for GRM/PCL or GRM/P4HB nanopapers followed previous reports.^3,4^ Briefly, PCL or P4HB pellets (50 mg) were dissolved in 150 mL of DMF at 60 °C for 1 h. Subsequently, 50 mg of GNP or rGO was dispersed in the PCL or P4HB solutions through pulsed sonication (5 s on, 5 s off, periodically) for 30 min at 30% of the maximum output power (500 W), using an ultrasonication probe (Sonics Vibracell VCX-750, Sonics and Materials Inc.) with a 13 mm Ti-alloy tip. The resulting suspension was then transferred into a filtration system equipped with a polyamide-supported membrane (0.45 μm nominal pore size, 47 mm diameter, Whatman) and left to filter overnight. After filtration, the sample, along with the filter, was placed in an oven to remove any residual solvent. The drying process involved holding at 70 °C for 2 h, followed by holding at 120 °C for an additional hour. Once dried, the nanopapers were carefully peeled from the filter and consolidated by applying a 6 ton load for 30 min at room temperature (RT). Additionally, selected nanopapers were extracted with toluene using a Soxhlet apparatus for 12 h to extract excess PCL and P4HB, then air-dried at room temperature for 48 h before further characterization. Extracted samples are identified in the following as SE for Soxhlet Extracted.

#### 2.2.2 Preparation of Nanopapers by Impregnation

The impregnation method consists of two steps. First, pristine GRM nanopapers were prepared by filtration from DMF suspensions, in the absence of polymer, and without mechanical pressing. In the second step, nanopapers were impregnated with a solution of PCL, P4HB or PGA and then mechanically consolidated. The desired PCL, P4HB or PGA amount (typically about 10% of the nanopaper’s mass) was dissolved in 5 mL of DMF (for PCL, P4HB) or HFIP (for PGA). Due to the high crystallinity of PGA at RT, which significantly limits its solubility in HFIP, PGA was first heated to 250 °C for 5 min and then rapidly cooled in liquid nitrogen. The amorphous PGA obtained was then dissolved in HFIP at 60 °C for 5 h. The polymer solutions were then added dropwise to the surface of the GRM nanopapers, which were placed on a hot plate and heated to 70 °C to allow the solvent to evaporate gradually. Finally, the samples were transferred to an oven at 120 °C for 1 h to remove any remaining solvent traces. After drying, the nanopapers were consolidated by applying a 6 ton load at RT for 30 min.

#### 2.2.3 Preparation of Nanopapers by Two-Step Filtration

Following the filtration procedure outlined in Section 2.2.1, GRM nanopaper was initially fabricated through the filtration process. The pristine, unpressed GRM nanopaper was placed on top of the PA filter membrane within the filtration device, as illustrated in Scheme 1. Separately, PCL or P4HB, in an amount equal to the mass of the GNP nanopaper, was dissolved in a DMF solution and heated with stirring at 60 °C for 1 h to ensure complete homogenization. The resulting solution was subsequently poured into the filtration device, allowing it to permeate through the GNP nanopaper under the influence of gravity. Upon completion of the filtration process, the nanopaper was carefully removed and subjected to a two-step drying protocol in an oven: first, at 70 °C for 2 h, followed by 120 °C for 1 h. Once dried, the nanopaper was pressed at room temperature under a 6 ton load for 30 min.

**Scheme 1:**
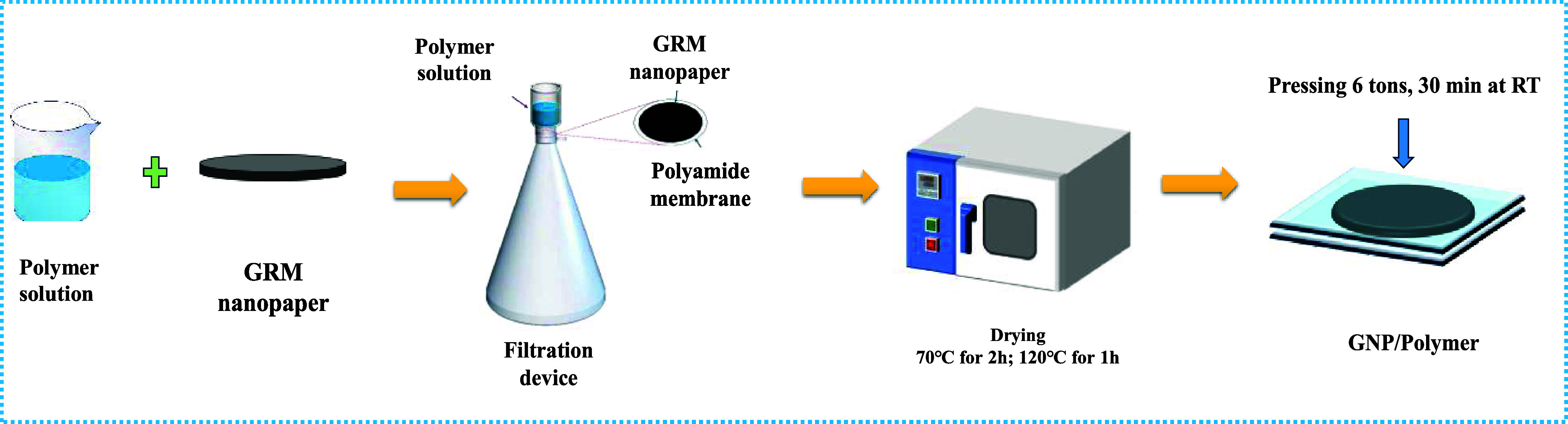
Preparation of Nanopaper via Two-Step Filtration

### 2.3 Characterization Methods

#### 2.3.1 Differential Scanning Calorimetry (DSC)

Nonisothermal DSC scans were performed using a DSC Q20 (TA Instruments, USA, calibrated with indium standards) or a DSC 8500 equipped with an Intracooler 3 cooling accessory (PerkinElmer, USA, calibrated with indium and zinc standards), respectively. All tests were performed by using hermetically sealed aluminum pans under an inert atmosphere (N_2_) with a heating and cooling rate of 20 °C/min.

Melting enthalpy values were calculated from the second DSC heating scans based on the actual polymer content in GRM nanopapers, determined by TGA analysis after DSC testing of the samples. The crystallinity (*X*_C_) of the polymer in different nanopapers was calculated by considering their real contents, following eq 1:

1 $$X_{C}\left(\%\right)=\frac{{\Delta H}_{m}}{{{\Delta H}_{m}^{0}}^{*}\Phi_{\mathrm{polymer}}}\times 100$$

where Δ*H*_m_ is the measured melting enthalpy, Φ_polymer_ is the polymer mass fraction in the nanopapers, and Δ*H*_m_^0^ is the melting enthalpy of 100% crystalline PCL (139.5 J/g),^22^ P4HB (151.0 J/g, according to calculations performed by the semiempirical group contribution theory by van Krevelen, detailed in Supporting Information) and PGA (139.0 J/g).^23,24^

#### 2.3.2 Successive Self-Nucleation and Annealing (SSA)

SSA was conducted on a PerkinElmer DSC 8500 equipped with an Intracooler 3 cooling accessory, according to the method established and reviewed by Müller et al.,^25–27^ utilizing fractionation windows of 2.5 °C for the highest-temperature endothermic peaks and 5 °C for intermediate and low-temperature melting peaks, with holding times of 5 min at each temperature. The testing procedure and methodology for fractionating these types of samples have been detailed in our previous reports.^3,4^

#### 2.3.3 Thermogravimetric Analysis (TGA)

TGA was performed using a Q5000 thermobalance (TA Instruments, USA) under a nitrogen (N_2_) atmosphere. The TGA specimens were retrieved from the crucible following nonisothermal DSC testing. Measurements were conducted at a heating rate of 10 °C/min over a temperature range of 50 to 600 °C.

#### 2.3.4 Wide-Angle X-ray Scattering (WAXS) and Grazing Incidence Wide-Angle X-ray Scattering (GIWAXS)

Measurements were performed on a Xeuss 2.0 SAXS/WAXS system (Xenocs SA, France) at RT. X-ray (wavelength = 1.5418 Å) was generated using a Cu Kα source (GeniX3D) at 50 kV and 0.6 mA. Scattered signals were measured by a semiconductor detector (Pilatus 300 K, DECTRIS, Switzerland) with a resolution of 487 × 619 pixels (pixel size 172 × 172 μm^2^). Each WAXS and GIWAXS pattern was recorded at an exposure time of 30 min. The 1D intensity profiles were integrated from background-corrected 2D WAXS patterns over an azimuth angle range of 0–90°.

#### 2.3.5 Variable Temperature Wide Angle X-ray Scattering (VT-WAXS)

VT-WAXS measurements were carried out at the BL16B1 beamline of the Shanghai Synchrotron Radiation Facility, using a Pilatus 900 K detector (Dectris, Switzerland) to collect two-dimensional diffraction patterns. The detector has a resolution of 981 × 1043 pixels with a pixel size of 172 × 172 μm^2^. The GNP:PCL (1:1), rGO:GNP (1:1), GNP:P4HB (1:1), and rGO:P4HB (1:1) samples obtained via the filtration method, as well as the GNP:PGA (10:1) sample prepared using the impregnation method, were heated at a rate of 5 °C/min on a Linkam THMS600 hot stage. Diffraction patterns were collected every 30 s, corresponding to one pattern every 2.5 °C. For the toluene-extracted samples (GNP:PCL 1:1, rGO:PCL 1:1), the heating rate on the hot stage was set to 10 °C/min. Diffraction patterns were collected every 6 s, corresponding to one pattern every 1 °C. The two-dimensional diffraction patterns were integrated using Foxtrot software, and one-dimensional diffraction curves were generated after subtracting the scattering background. Before testing, all samples were crystallized under controlled conditions: the temperature was raised to 200 °C to erase the thermal history, followed by cooling at a rate of 10 °C/min to −20 °C and an isothermal step for 3 min. The sample prepared with this method is, therefore, consistent with the second heating scan in the conventional DSC test.

## 3 Results and Discussion

### 3.1 Crystallization of PCL, P4HB and PGA within GRM Nanopapers

#### 3.1.1 Crystal Structure of PCL, P4HB and PGA

PCL, P4HB, and PGA belong to a class of semicrystalline polyesters with similar chemical repeating units, differing by the number of methylene groups between the ester groups, as illustrated in Figure 1a. Figure 1d,e displays the indexed WAXS patterns for the main reflections of the three samples and all three polymers mainly crystallized into orthorhombic α crystals.^28–30^ The unit cell parameters for PCL are *a* = 0.748 nm, *b* = 0.498 nm, and *c* = 1.726 nm;^29,31^ for P4HB, they are *a* = 0.775 nm, *b* = 0.477 nm, and *c* = 1.199 nm;^28,32,33^ and for PGA, they are *a* = 0.522 nm, *b* = 0.619 nm, and *c* = 0.702 nm.^30^ Among them, PGA possesses the shortest and most regular main chain, adopting an almost completely all-*trans* (planar zigzag) conformation in the crystal.^34–36^ Its tightly packed molecular chains and high crystal density contribute to its excellent mechanical properties and high melting point, as shown in Figure 1c and Table 1. In contrast, PCL contains multiple methylene groups (—(CH_2_)_5_—) and adopts in the crystalline state, planar zigzag, all-*trans* conformation.^37,38^ P4HB, with an intermediate methylene chain (—(CH_2_)_3_—), adopts all-*trans* (planar zigzag, slightly distorted) conformation.^39^ The melting points are in the order *T*_m,PGA_ ≫ *T*_m,P4HB_ > *T*_m,PCL_ (Figure 1c), reflecting chain segment flexibility, PGA < P4HB ≪ PCL. The crystallinities of the three polymers, calculated from WAXS measurements, are comparable in PCL and P4HB, and the PGA is highest (Table 1).

**Figure 1:**
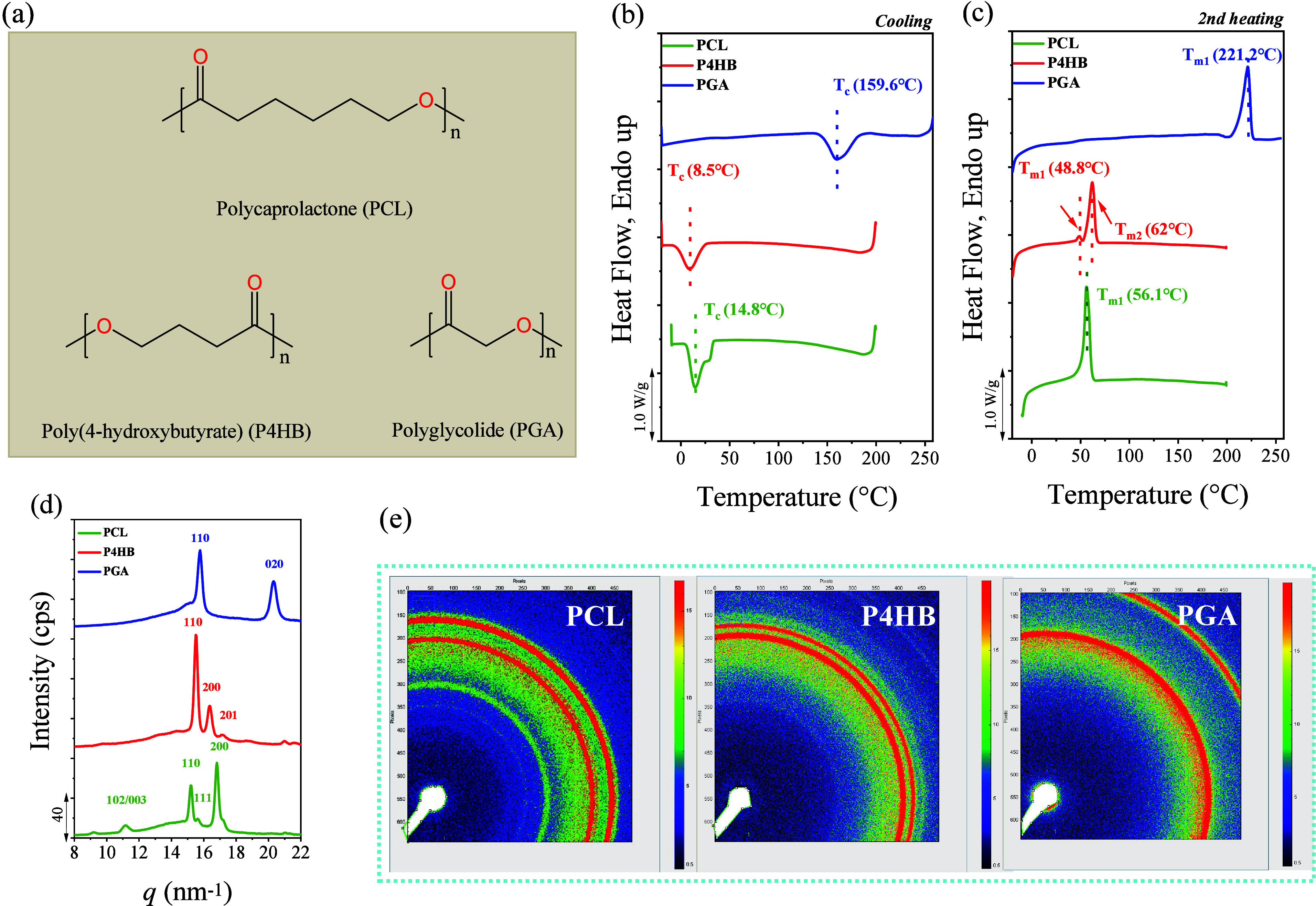
General characterization of PCL, P4HB and PGA: (a) Chemical structures, (b) DSC cooling scans, (c) Second heating DSC scans, (d) WAXS diffractograms, (e) 2D WAXS patterns.

**Table 1:** Thermal Properties of the Employed PCL, P4HB, and PGA^a^

	*T*_m_ (°C)					
polymer	*T*_m1_	*T*_m2_	*T*_c_ (°C)	Δ*H*_m_ (J/g)	crystallinity (%) (DSC)	crystallinity (%) (WAXS)
PCL	56.1		14.8	61.5	44	45
P4HB	48.8	62.0	8.5	36.3	24	41
PGA	221.2		159.6	58.6	42	52

a *T*_m_, Δ*H*_m_ and crystallinity are calculated from the DSC second heating curve, the heating/cooling rates employed were 10 °C/min, and the values of the melting enthalpy of 100% crystalline samples employed were: PCL (139.5 J/g), P4HB (151.0 J/g) and PGA (139.0 J/g). The crystallinity of P4HB was calculated by WAXS fitting (Figure S1).

#### 3.1.2 DSC Analysis of Nanopapers

Since PCL and P4HB have similar melting points (Figure 1d and Table 1) and good solubility in DMF at 60 °C, the filtration method could be applied for the preparation of GRM/polymer nanopapers. This method was not applicable to PGA, which could not be added to the GRM suspension for the direct filtration, owing to insufficient solubility in DMF. The DSC curves for different GRM-based nanopapers (GNP and rGO) containing PCL and P4HB, prepared using the filtration method, are compared in Figure 2 and Table 2.

**Figure 2:**
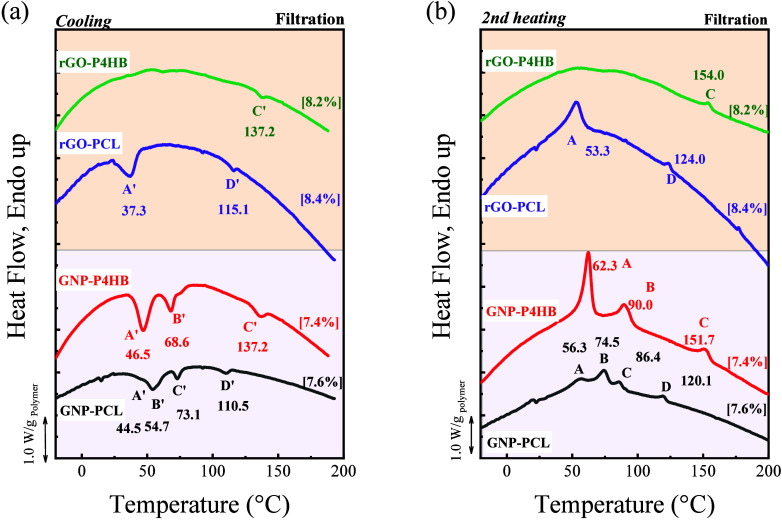
DSC curves of PCL and P4HB nanopapers on GNP or rGO obtained by the filtration method: (a) Cooling scans, (b) Second heating scans. In brackets are the polymer contents from TGA (see Figure S2).

**Table 2:** Summary of the DSC Results of the Nanopapers Prepared by Different Methods

		*T*_m_ (°C)				*T*_c_ (°C)			
material	method	*T*_m1_	*T*_m2_	*T*_m3_	*T*_m4_	*T*_c1_	*T*_c2_	*T*_c3_	*T*_c4_
GNP:PCL 1:1	filtration	56.3	74.5	86.4	120.1	44.5	54.7	73.1	110.5
GNP:P4HB 1:1		62.3	90.0	151.7		46.5	68.6	137.2	
rGO:PCL 1:1		53.3			124.0	37.3			115.1
rGO:P4HB 1:1				154.0				137.2	
GNP:PCL 10:1	impregnation	56.0	74.5	86.4	116.2	45.0	56.0	74.1	107.4
GNP:P4HB 10:1		51.2	62.6	87.7		24.5	47.8	65.7	
GNP:PGA 10:1		216.5	241.7			175.1			
rGO:PCL 10:1		56.7				38.7			
rGO:P4HB 10:1		62.6		154.9		43.1		140.8	
rGO:PGA 10:1		216.5							

The thermal behavior of the GNP-PCL nanopaper was previously reported, showing distinct cooling and melting first-order transitions. In the case of the melting endotherms (Figure 2b), the different melting transitions are Peak A, ca. 56 °C, assigned to unoriented PCL, peak B, ca. 75 °C, assigned to oriented PCL, and peaks C and D, ca. 86 and 120 °C, tentatively assigned to prefreezing crystalline layers of PCL crystals within the nanopaper.^3,4^ Indeed, the A peak corresponds to the melting of the pristine PCL (Figure 1c) and the B peak may derive from the presence of thicker lamellae as a consequence of polymer nucleation onto the GNP, which is commonly reported in the literature. However, the C and D peaks are observed above the equilibrium melting temperature of PCL, indicating a peculiar condition for the stabilization of the polymer chains’ organization at high temperatures. The formation of highly stable prefrozen crystals of PCL onto the surface of HOPG, as previously reported in the literature,^15,17^ provides a likely explanation for the physical origin of peak C, taking into account of the homologous structure of GNP and HOPG. By extension of this concept, the presence of peak D appears justified in case where prefrozen crystals form between two GNP, so that stabilization to higher temperatures may be possible. Indeed, the melting temperature of prefrozen crystals depends on the interfacial energy balance, where the strong interaction between the PCL crystal and GNP plays an important role. Should prefrozen crystals form in the gallery between two GNP, the same PCL crystal/GNP interaction would act on both interfaces (top and bottom) for the same crystal with the two graphite nanoplates, potentially maximizing the effect of stabilization at high temperature. It is also worth noting that each endothermic transition in the DSC heating plots corresponds to a peak in the cooling plots. In particular, exothermic peaks on cooling plots, corresponding to the C and D peaks, occur at temperatures comparable (C’, 73.1 °C) or higher (D’, 110.5 °C) than the equilibrium melting temperature for PCL,^4,40^ thus well above the crystallization temperatures for the main polymer fraction (A’ and B’ peaks). This allows ruling out the alternative interpretation of C and D endothermic peaks as the disordering of a kinetically trapped metastable structure caused by strong adsorption onto the GNP. In rGO-PCL nanopaper, only peaks A and D are visible, which was attributed to the morphological and chemical defects in rGO compared to GNP.^4^

Despite its structural similarity to PCL, P4HB exhibits a different thermal behavior when embedded in both GNP and rGO nanopapers. P4HB exhibits three melting peaks (namely peak A at 62.3 °C, peak B at 90.0 °C, and peak C at 151.7 °C, see Figure 2b and Table 2,) on GNP, with each melting peak having a corresponding crystallization peak, as shown in Figure 2a. Compared to the DSC curve of neat P4HB (Figure 1c), the first peak clearly corresponds to the melting of conventional unoriented P4HB crystals, whereas the two additional peaks at higher temperatures are originated as a consequence of the interaction with GNP flakes. The peak B of P4HB at 90 °C is quite broad and may correspond to the peaks B and/or C of PCL. It is worth mentioning that the equilibrium melting temperature for P4HB has been reported as 79.9 °C,^28^ suggesting the second peak observed for P4HB may not be explained simply by the higher stability of oriented crystals. The peak C of P4HB (151.7 °C) is approximately 30 °C higher than the D peak for PCL, suggesting that the high-temperature signal previously observed in PCL as peak D, has an analogous counterpart in P4HB. In rGO nanopapers, P4HB exhibits only weak and broad traces of melting signals for either unoriented P4HB or other peaks in the range of 90 °C (peak B). Conversely, a peak at 153.6 °C (peak C) is clearly visible with an intensity comparable to that exhibited by the GNP-PCL nanopaper. This suggests that the majority of P4HB is not able to crystallize during cooling under these conditions (only weak traces of crystallization peaks are visible in the range between 70 and 40 °C, Figure 2a), but the organization responsible for the high-temperature signal is clearly obtained in similar conditions.

To investigate the effect of the preparation method and enlarge the polymer comparison to PGA, nanopapers prepared by impregnation were also studied. Figure 3 and Table 2 compare the DSC plots for GRM nanopapers containing PCL, P4HB, and PGA, respectively, prepared by using the impregnation method. For PCL, peaks A, B, and C are clearly observed in GNP nanopapers (Figure 3b), with only weak traces of the D peak. This was previously explained by the limited time for the PCL to absorb on GNP during the impregnation process. Similarly, for P4HB, the DSC curve reveals two melting peaks A and B (62.6 and 87.7 °C) on GNP. For PGA in GNP nanopapers, the main melting signal is visible at 216.5 °C (labeled peak A), which is only slightly lower than for the pristine PGA (Figure 1c), with an additional peak at 241.7 °C (labeled peak B). It is worth mentioning that all the endothermic signals described on heating found a one-to-one correlation with corresponding exothermic signals in the cooling plots, expect for PGA nanopapers, where the cooling peak corresponding to the melting of the highest stability crystals is not clearly observable. In addition, in Figure 3b, it can be observed that P4HB has a low exothermic peak at 24.5 °C, which does not exist in the filtration method, confirming that the impregnation method reduces the nucleation efficiency of GNPs on P4HB. In rGO nanopapers, during heating, for PCL and PGA, only the peak for unoriented crystals is visible, while P4HB shows traces of a signal at ∼155 °C (peak C), in addition to the peak for conventional unoriented crystals. It therefore appears that the higher surface area and greater defect density of rGO compared to GNP limit the crystallinity of the polymers, so only the most intense signal (unoriented polymer crystals) is observed.

**Figure 3:**
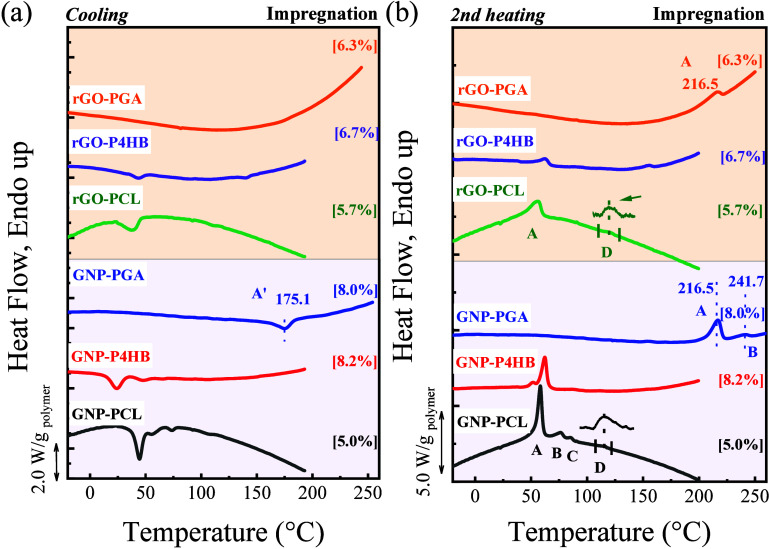
DSC curves of PCL and P4HB nanopapers on GNP or rGO obtained by the impregnation method. (a) cooling scans, (b) second heating scans. Insets show a magnification of the trace signals. In brackets are the polymer contents from TGA (Figure S2).

To further investigate the role of polymer adsorption in the organization of polymer crystals, a new method was explored (section 2.2.3), by the filtration of a polymer solution through a preformed nanopaper of pristine GNP or RGO. This method, referred to as “two-step filtration”, yielded low polymer fractions (2.5∼3.5 wt %) in nanopapers. Interestingly, both PCL and P4HB solutions filtered through GNP or rGO nanopapers exhibit only peaks D and C, respectively (Figure S3). This fits the results of the nanopapers prepared by the conventional filtration method and subsequently underwent polymer extraction in Soxhlet. This proves that polymer adsorption during filtration through the nanopaper is required for the formation of structures corresponding to the highest DSC peak.

### 3.2 Structural Investigation of the Crystal Populations of PCL, P4HB and PGA within GRM Nanopapers

To investigate the basic properties of each melting peak of the crystal populations in PCL and P4HB nanopapers, DSC at different heating rates (Figure S6) was carried out. As the heating rate increases, peaks C and D of PCL shift significantly toward higher temperatures, while peaks A and B increase only slightly with heating rate (Figure 4a). P4HB nanopapers also showed similar results: peaks A and B are nearly heating-rate-independent, whereas peak C shows greater heating-rate dependence (Figure 4b). The strong dependence of high-temperature transitions may suggest a kinetically controlled process, analogous to desorption. This appears to support the occurrence of melting immediately after the desorption of crystals from the GRM surface.

**Figure 4:**
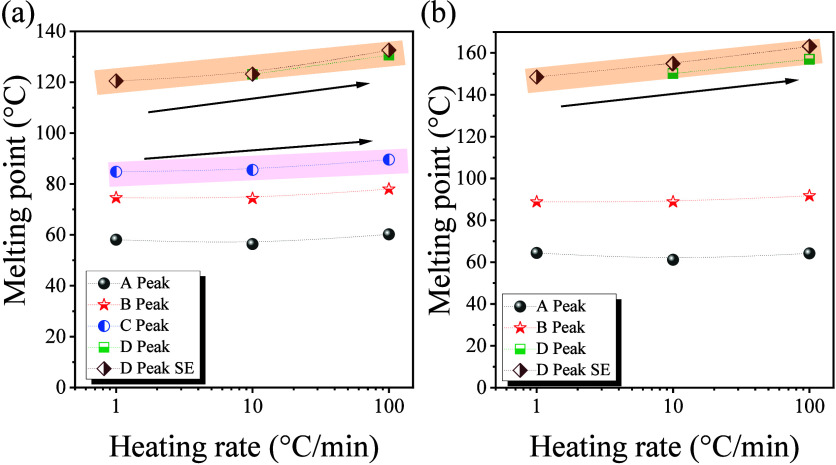
Heating rate dependence of melting points corresponding to the observed different crystal populations for nanopapers prepared by filtration (a) GNP-PCL and (b) GNP-P4HB.

Based on the differences in the organization of high stability crystal populations in the nanopapers prepared with the different polymers, a structural investigation was carried out by means of successive self-nucleation and annealing and X-ray diffraction.

#### 3.2.1 SSA

SSA was performed to investigate the possibility of thermally fractionating the different crystalline populations, which may be indirectly related to the nature of interactions between polymer chains and inorganic particles, as previously observed for PCL.^3,4^ Figure 5 shows the SSA results for PCL and P4HB embedded in GNP-based nanopapers obtained by either filtration or impregnation. The final heating curves after SSA exhibit a series of characteristic peaks that indicate the melting of different crystalline thermal fractions. Consistent with previous findings, the highly stable structures formed in nanopapers prepared via either filtration or impregnation methods displayed variations in relative intensity, further confirming the nonisothermal DSC results shown in Figures 2 and 3. Notably, the bulk melting peaks of PCL and P4HB in nanopapers obtained through impregnation were more pronounced than those in nanopapers prepared by filtration. This suggests that the primary signal in the impregnated nanopapers corresponds to weaker polymer-GRM interactions. In previous studies, fractionation peaks distinct from those of PCL have been systematically analyzed,.^4^ In particular, PCL showed fractionation for both the unoriented and oriented crystalline populations, observable as a series of melting peaks in the temperature range 22 to 66.0 °C (peak A) and 66 to 80.0 °C (peak B), respectively. Conversely, no evidence for fractionation of peaks previously identified as C and D has been found, as previously reported. Similarly, crystalline populations for both unoriented and oriented P4HB crystals are clearly thermally fractionated into a series of melting peaks in the ranges 22.0 to 72.0 °C (peak A) and 72.0 to 100.0 °C (peak B), while the high-temperature signal remains consistent with conventional DSC. The series of peaks below 72 °C closely resembles that of neat SSA-fractionated P4HB (Figure S7) in both position and relative intensities, confirming assignment to unoriented P4HB.

**Figure 5:**
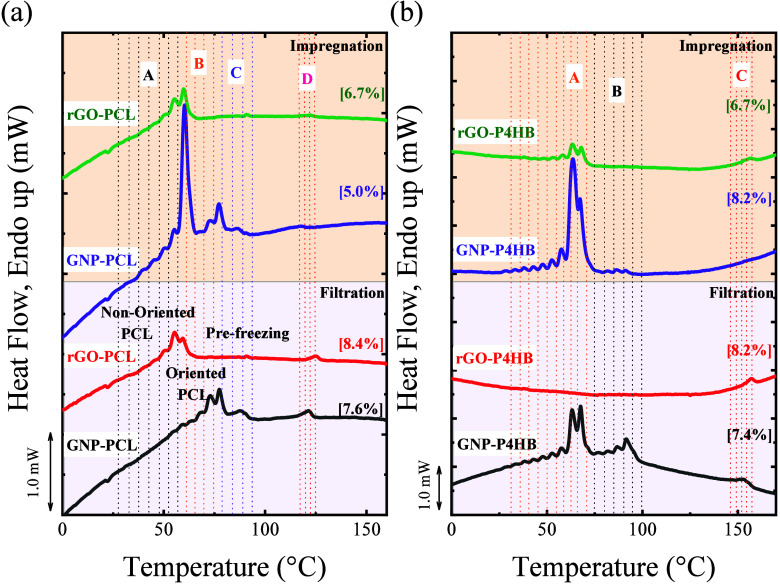
Final DSC heating curves after SSA of PCL and P4HB nanopapers prepared by different methods and different GRMs: (a) PCL, (b) P4HB. In brackets are the polymer contents from TGA (Figure S2).

The series of melting peaks at higher temperatures (ca. 72.0 to 100.0 °C) is not observed in pristine P4HB (Figure S7), and it is clearly visible only in GNP nanopapers. The highest melting peak of this group is above the reported equilibrium melting temperature of P4HB (79.9 °C).^28^ The observed SSA fractionation suggests that the origin of this family of signals corresponds to the presence of oriented P4HB crystals, in analogy with the findings for PCL, rather than prefrozen crystals. Regarding the unfractionated melting peak at 151.7 °C (peak C), although its exact origin remains unclear, it is considered similar to the behavior observed in PCL and is attributed to the formation of a highly stable structure due to strong interactions between P4HB and GNP flakes. The SSA results for P4HB on rGO, reveal only a single peak at 151.7 °C (peak C), aligning with the traditional nonisothermal test results. The absence of a 90 °C peak suggests that the structural defects in rGO negatively impact the formation of oriented P4HB, further supporting the notion that rGO imposes stronger constraints on P4HB crystallization.

SSA procedure was also applied to PGA/GNP nanopaper (Figure S8), showing traces of fractionation of peak A signal, whereas peak B was found to be clearly visible at 248 °C and unfractionated. Based on this result and the reported equilibrium melting temperature for PGA is 231.5 °C,^41^ it appears reasonable to speculate that the highest endothermic peak of PGA (peak B) in the DSC results (Figure 3) may also correspond a prefrozen structure.

### 3.3 WAXS and GIWAXS

X-ray scattering experiments were carried out to investigate the orientation and stability of the crystals. A distinct orientation structure in nanopapers by GIWAXS were previously observed for PCL in both GNP and rGO nanopapers.^4^ The preferential orientation in nanopapers containing PCL and P4HB were studied as well. 2D WAXS images (Figure 6a,b) with isotropic signals are observed by transmission mode, as expected, while strong anisotropic signals are observed in GRM, PCL and P4HB in GIWAXS mode (Figure 6c,d).

**Figure 6:**
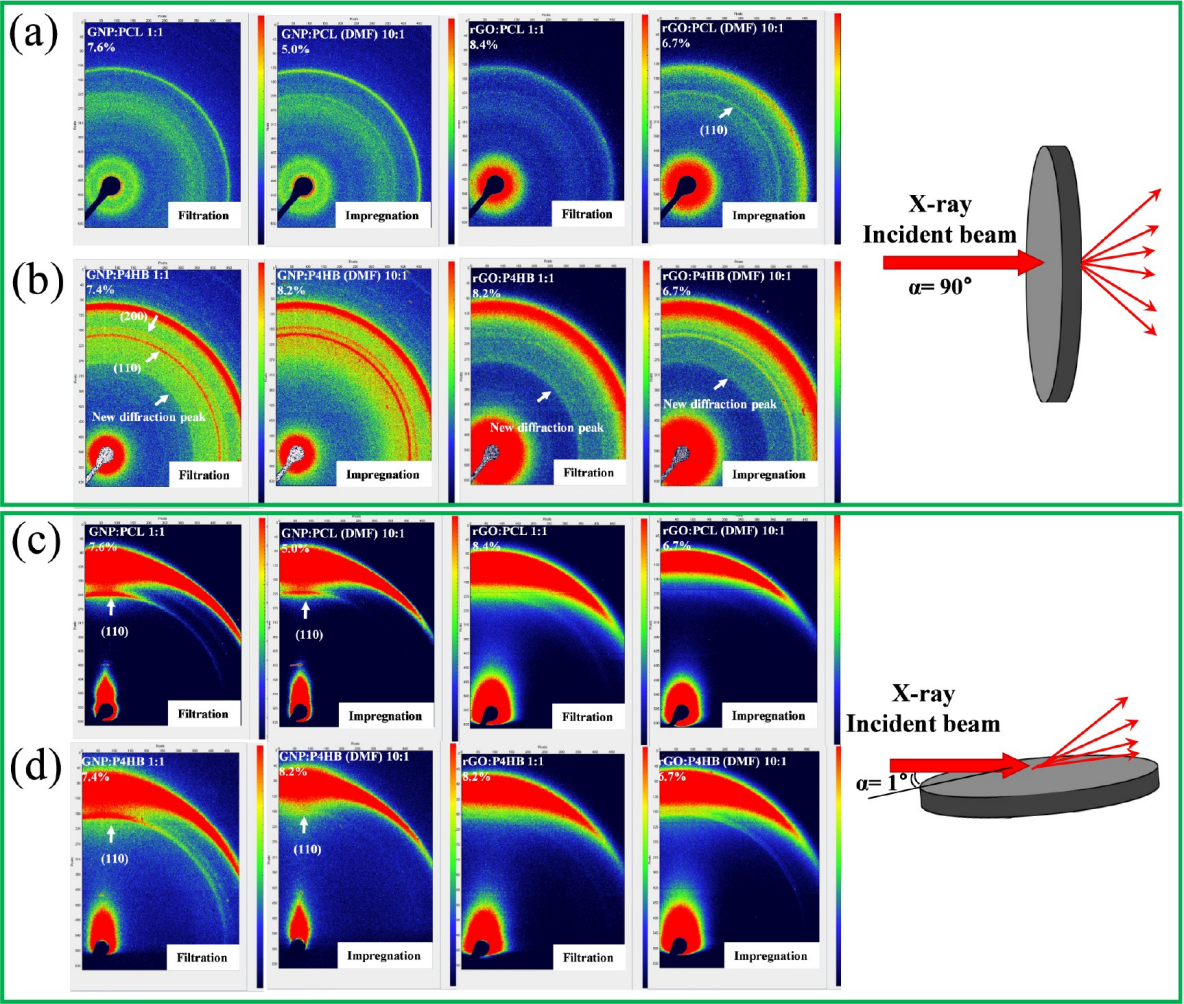
2D WAXS images of PCL and P4HB nanopapers prepared by different preparation methods with different GRMs. Transmission modes (a) PCL and (b) P4HB. GIWAXS modes (c) PCL and (d) P4HB.

For both nanopapers obtained by filtration and impregnation, the intensity profiles obtained from azimuthal integration (0–90°) of the transmission mode are shown in Figure S9, highlighting the presence of (110) and (200) reflections of PCL α crystals and P4HB α crystals. In GIWAXS mode, only (110) reflection of PCL and P4HB is observed (Figure 6c, d, and S11), suggesting a certain preferential orientation of polymer crystals, depending on the preparation method and the GRM type.

Interestingly, P4HB displays a new broad diffraction signal that emerges at *q* = 12.2 nm^–1^(Figure 7b and S10b) in transmission mode, in both GNP and rGO nanopapers, corresponding to a distance of 5.15 Å. This signal is not associated with any known P4HB crystals, as it was also not observed for pristine P4HB (Figure 1d, e) and GRM-PCL. The same signal is not observed in the GIWAXS configuration (Figure S11), evidencing that this signal is direction-dependent. This peak appears to originate from a regular arrangement of P4HB chains along the GRM basal plane, possibly explained by a mesomorphic structure adsorbed on the surface of GRM.^42–44^

**Figure 7:**
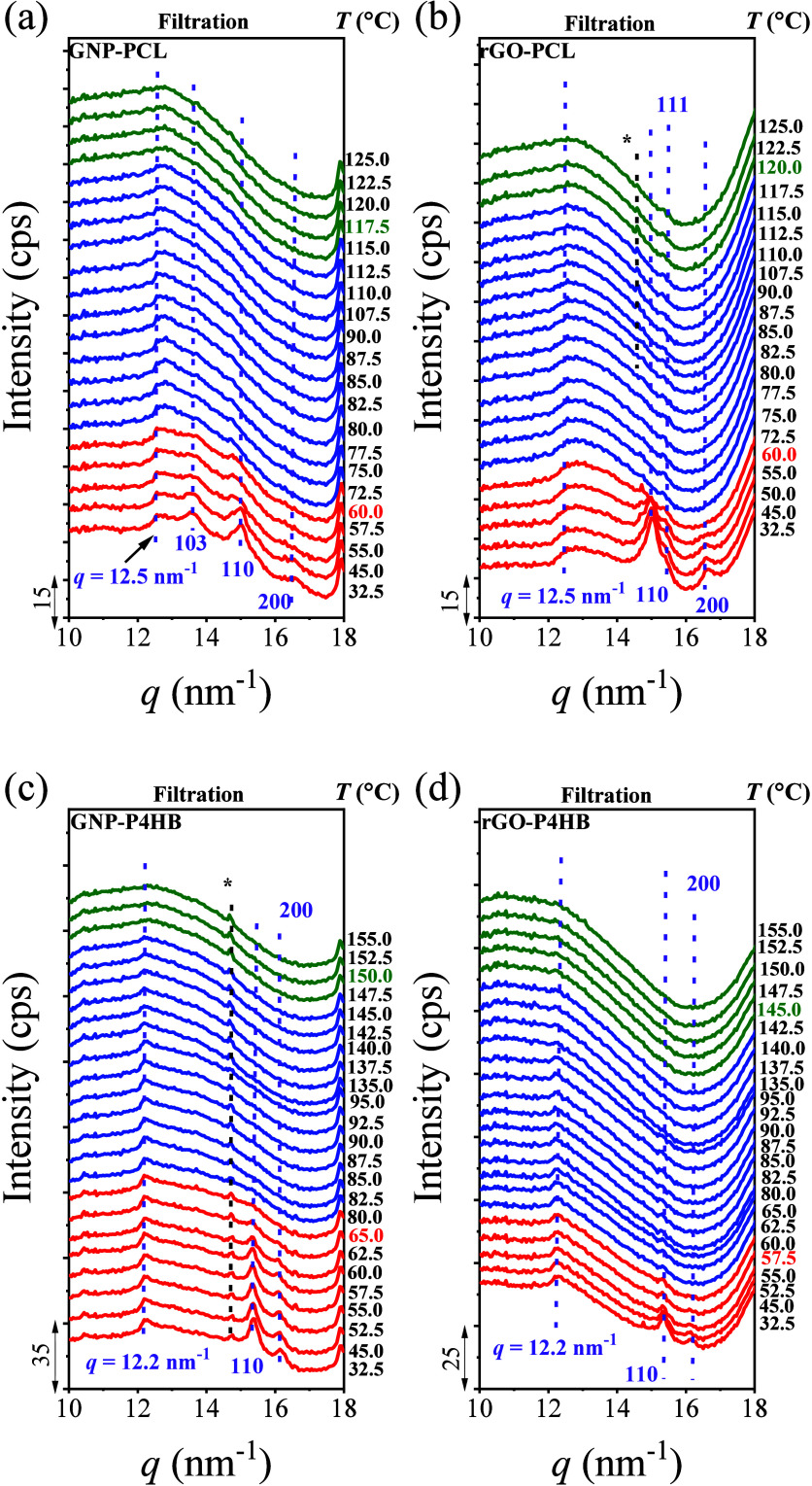
WAXS patterns taken during heating at the selected temperatures for (a) GNP-PCL, (b) rGO-PCL, (c) GNP-P4HB, and (d) rGO-P4HB. The peaks marked with * in the figure do not belong to polymers or graphene, but are assigned to impurities.

WAXS diffraction patterns of PGA/GRM nanopapers show that only a weak PGA diffraction peak is observed at *q* = 15.7 nm^–1^in transmission mode, assigned to the (110) plane, while in the GIWAXS, no diffraction peak is detected (Figure S12). This weaker signals, compared to PCL and P4HB in nanopapers obtained by impregnation, may be attributed to the high evaporation rate of the solvent for PGA, as DMF used for PCL and P4HB has a much higher boiling point compared to HFIP used for PGA. .

### 3.4 Variable Temperature WAXS

To investigate the thermal stability of crystalline structures of PCL and P4HB in nanopapers, X-ray scattering patterns were acquired on heating. Figure 7 presents the transmission results of nanopapers containing PCL or P4HB, based on GNP or rGO, prepared by filtration.

Figure 7a and b show the changes in the diffraction signals of PCL on GNP and rGO over a selected temperature range from 30 to 125 °C. As reported above (Figure 1), the crystal diffraction peaks of PCL in GNP nanopapers are observed at *q* = 13.6, 15.0, and 16.5 nm^–1^, corresponding to the crystal planes of the (103), (110), and (200), respectively, which disappear at around 60 °C. In rGO nanopapers, the crystal diffraction peaks appear at *q* = 15.0, 15.4, and 16.5 nm^–1^, corresponding to the (110), (111), and (200) planes, which also disappear at around 60 °C. These results indicate that on both GNP and rGO, the primary melting temperature in DSC (peak A) corresponding to the pristine PCL, i.e., nonoriented crystals. No significant changes in the WAXS patterns are visible in the temperature range covering peak B and peak C in DSC. Similar findings are observed for P4HB nanopapers, as shown in Figures 7c, d. The main crystal diffraction peaks of P4HB on GNP and rGO are located at *q* = 15.4 and 16.2 nm^–1^, corresponding to the (110) and (200) crystal planes, respectively. The disappearance temperatures for such signals occur at approximately 65.0 °C (peak A) for GNP-P4HB, in agreement with the melting of the main peaks in DSC (Figure 2b) and 57.5 °C (peak A) for rGO-P4HB. The latter confirms the existence of unoriented crystals of P4HB, which were hardly detected in DSC.

The absence of clear diffraction signals in PCL and P4HB above the melting temperature of their main fraction (peak A) is compatible with the orientation of low fractions of the remaining crystals, whose presence is well-supported by both conventional DSC and SSA, as discussed above. Indeed, the most intense signal from 110 planes of remaining crystals may not be observed, taking into account its preferential orientation parallel to the GRM basal plane (Figure 6c,d).

Besides the peaks assigned to the well-known polymer crystalline planes, the thermal stability of the signals in the low *q* range in nanopapers containing PCL or P4HB was also analyzed. For PCL, in both GNP and rGO nanopapers, a novel diffraction peak signal at *q* = 12.5 nm^–1^ is clearly visible up to about 115 °C. It is worth noting that this signal was not observable in conventional WAXS (Figure S10a). The disappearance temperature of this diffraction peak aligns with the highest melting temperature of the D peak observed in the DSC measurements. For P4HB, a similar signal is observed at *q* = 12.2 nm^–1^ (Figure 7c,d), which is retained up to approx 150 °C, which again corresponds to the highest-temperature endothermic signal (peak C) in the DSC results. These results demonstrate that the highest-temperature transition observed in both PCL and P4HB in nanopapers indeed corresponds to an order–disorder transition.

In addition, the VT-WAXS results of nanopapers impregnated with PCL, P4HB and PGA (Figure S13) show that the new diffraction peak (*q* = 12.2–12.5 nm^–1^ signals) is visible for PCL and invisible in P4HB, in agreement with the absence of clear high-temperature peak in DSC (Figure 3b), while PGA only shows the diffraction signal of the (110) plane.

To further investigate the nature of the high-temperature transition, GNP-PCL and rGO-PCL nanopapers were selected for analysis after the extraction of the polymer. This was previously reported to efficiently remove most PCL from the nanopapers, thereby eliminating the main melting peaks in DSC. However, it was also reported that a small fraction of PCL (2.5∼4.0% wt., Figure S4) is retained after extraction, which was interestingly associated with the retention of the highest-temperature transition in DSC (peak D, Figure S3a,b). The VT-WAXS results for extracted GNP-PCL and rGO-PCL nanopapers (Figure 8a, b) reveal only one clear signal at *q* = 12.5 nm^–1^, confirming that PCL organized in conventional crystals was indeed removed. Most importantly, that signal disappears at about 120 °C, thus matching the temperature of the D peak (Figure S14). This confirms that the retained PCL is responsible for the D peak and suggests that its retention is related to a particularly stable ordered arrangement of chains onto the GRM, which is lost only above 120 °C.

**Figure 8:**
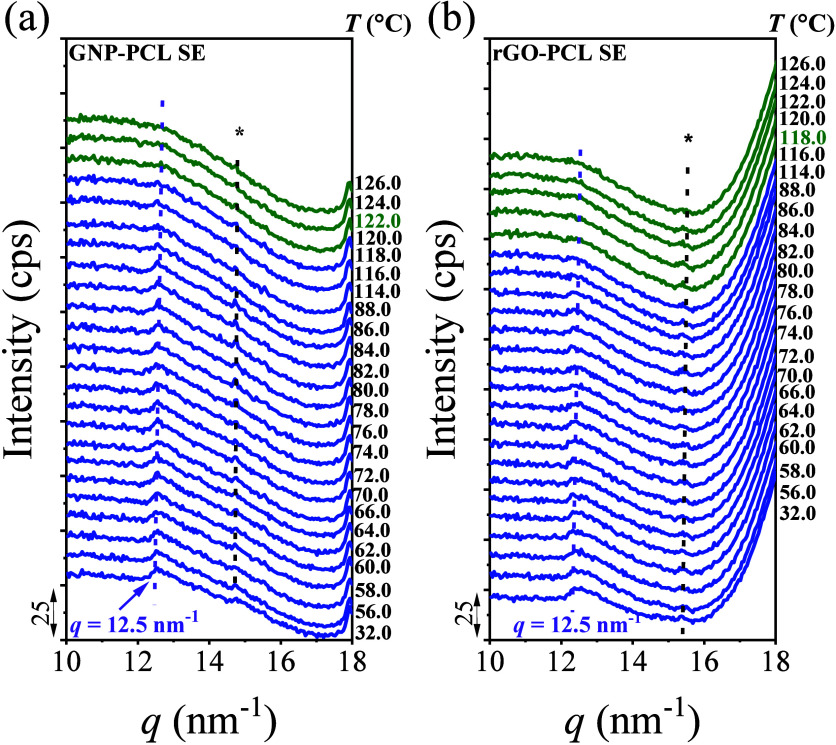
WAXS patterns taken during heating at the selected temperatures for (b) GNP-PCL SE and (c) rGO-PCL SE. The peaks marked with * in the figure do not belong to polymers or graphene, but are assigned to impurities.

## 4 Conclusions

GRM nanopapers embedding limited amounts of biopolyesters with variable methylene chain lengths between ester groups were studied to investigate the influence of the GRM particles on polymer crystallization. Building on previous research, this work demonstrates that the occurrence of multiple crystalline populations for polymer within the GRM nanopapers is not unique to PCL, and it is observed also for P4HB. PGA was also addressed to extend and generalize the phenomenological study in this work; despite the limitations in the nanopaper processing due to its poor solubility in conventional solvents, evidence of at least two crystalline populations was obtained for PGA.

Besides the well-known nucleation activity of GRM, the interaction between the macromolecules with the surface of GNP and rGO appears to drive the formation of highly stable crystals, in addition to conventional polymer crystals and oriented crystals obtained from heterogeneous nucleation onto GRM (A and B peaks, respectively). For both PCL and PH4B, first-order transition signals well above the equilibrium melting temperatures were clearly observed by DSC. However, two endothermic peaks (ca. 85 and 120 °C for C and D peaks, respectively) were observed on heating for PCL above *T*_m_^0^, whereas only one signal was clearly visible for P4HB at *ca*. 150 °C (C peak) under the same conditions. This is possibly explained by an overlap of different P4HB signals into a relatively broad band at ca. 90 °C, which may account for both the melting of oriented crystals and the signal corresponding to the PCL C peak. Besides, the highest-temperature signal for P4HB is significantly higher than that of the PCL counterpart, both in absolute value and in the temperature difference relative to their respective *T*_m_^0^ values. This suggests that the strong interaction between the P4HB chains (and, to a lesser extent, the PCL chains) and the GRM surface is driving the regular organization of the chains on the surface and preserving this organization up to a temperature well above the melting point of ideal polymer crystals. Further insights into the nature of these high-temperature signals were obtained by successive self-nucleation and annealing (SSA) analyses, which revealed that peaks C and D cannot be resolved through fractionation, confirming that the polymer chains in those structures are strongly constrained by the effect of their strong interaction with the GRM surface. Additionally, variable-temperature WAXS analyses unveiled the existence of a specific WAXS signal disappearing at ca. 120 °C for PCL and 150 °C for P4HB, demonstrating for the first time that the high-temperature transitions are correlated with an order–disorder transition occurring once the temperature is sufficiently high to overcome the interactions of polymer chains on the surface of GRM.

These results may be interpreted as the formation of prefrozen crystalline structures, generalizing the previous experimental studies on different polymers on the surface of highly oriented pyrolytic graphite to three-dimensional networks of graphene-related materials. Indeed, the thermodynamic principles driving the formation of thin crystals on the surface of highly oriented pyrolytic graphite and their stabilization up to temperatures above *T*_m_^0^ may be applied within nanopapers, where prefrozen crystals may be stabilized not only on surfaces but also into the galleries between GRM layers.

Controlling the crystalline structure and thermal stability of polymer chains within nanoparticle networks provides a compelling method for developing new hybrid organic–inorganic materials for thermal and thermomechanical uses.

## Supplementary Material



## Notes

Additional characterization of polymers and nanopapers; Calculation of crystallization enthalpies; WAXS, TGA, and DSC data (PDF)

## References

[1] Volchko N. W., Rutledge G. C. (2023). Heterogeneous nucleation of high-density polyethylene
crystals on graphene within microdomains. Macromolecules.

[2] Colonna S., Pérez-Camargo R. A., Chen H., Liu G., Wang D., Müller A. J., Saracco G., Fina A. (2017). Supernucleation
and orientation of poly (butylene terephthalate) crystals in nanocomposites
containing highly reduced graphene oxide. Macromolecules.

[3] Li K., Battegazzore D., Perez-Camargo R. A., Liu G., Monticelli O., Muller A. J., Fina A. (2021). Polycaprolactone Adsorption and Nucleation
onto Graphite Nanoplates for Highly Flexible, Thermally Conductive,
and Thermomechanically Stiff Nanopapers. ACS
Appl. Mater. Interfaces.

[4] Zhao H., Pérez-Camargo R. A., Damonte G., Armandi M., Monticelli O., Liu G., Müller A. J., Fina A. (2025). Crystallization of
Polycaprolactone within Nanopapers Based on Graphene-Related
Materials. Macromolecules.

[5] Tariq M., Dolynchuk O., Thurn-Albrecht T. (2019). Effect of
Substrate Interaction on
Thermodynamics of Prefreezing. Macromolecules.

[6] Dolynchuk O., Schmode P., Fischer M., Thelakkat M., Thurn-Albrecht T. (2021). Elucidating the Effect of Interfacial Interactions
on Crystal Orientations in Thin Films of Polythiophenes. Macromolecules.

[7] Dolynchuk O., Kahl R. T., Meichsner F., Much A. J., Pechevystyi A., Averkova A., Erhardt A., Thelakkat M., Thurn-Albrecht T. (2024). Controlling Crystal Orientation in
Films of Conjugated
Polymers by Tuning the Surface Energy. Macromolecules.

[8] Beuguel Q., Boyer S. A., Settipani D., Monge G., Haudin J. M., Vergnes B., Peuvrel-Disdier E. (2018). Crystallization behavior of polypropylene/graphene
nanoplatelets composites. Polymer Crystallization.

[9] Hua L., Kai W., Inoue Y. (2007). Synthesis
and characterization of poly (ϵ-caprolactone)–graphite
oxide composites. J. Appl. Polym. Sci..

[10] Müller A. J., Arnal M. L., Trujillo M., Lorenzo A. T. (2011). Super-nucleation
in nanocomposites and confinement effects on the crystallizable components
within block copolymers, miktoarm star copolymers and nanocomposites. Eur. Polym. J..

[11] Trujillo M., Arnal M., Müller A. J., Laredo E., Bredeau S., Bonduel D., Dubois P. (2007). Thermal and
morphological characterization
of nanocomposites prepared by in-situ polymerization of high-density
polyethylene on carbon nanotubes. Macromolecules.

[12] Priftis D., Sakellariou G., Hadjichristidis N., Penott E. K., Lorenzo A. T., Müller A. J. (2009). Surface
modification of multiwalled carbon nanotubes
with biocompatible polymers via ring opening and living anionic surface
initiated polymerization. Kinetics and crystallization behavior. J. Polym. Sci., Part A: Polym. Chem..

[13] Trujillo M., Arnal M., Müller A. J., Bredeau S., Bonduel D., Dubois P., Hamley I., Castelletto V. (2008). Thermal fractionation
and isothermal crystallization of polyethylene nanocomposites prepared
by in situ polymerization. Macromolecules.

[14] Löhmann A.-K., Henze T., Thurn-Albrecht T. (2014). Direct observation of prefreezing
at the interface melt–solid in polymer crystallization. Proc. Natl. Acad. Sci. U. S. A..

[15] Tariq M., Dolynchuk O., Thurn-Albrecht T. (2020). Independent Variation of Transition
Temperature and Prefrozen Layer Thickness at the Prefreezing Transition. J. Phys. Chem. C.

[16] Dolynchuk O., Tariq M., Thurn-Albrecht T. (2019). Phenomenological
Theory of First-Order
Prefreezing. J. Phys. Chem. Lett..

[17] Flieger A.-K., Schulz M., Thurn-Albrecht T. (2018). Interface-Induced Crystallization
of Polycaprolactone on Graphite via First-Order Prewetting of the
Crystalline Phase. Macromolecules.

[18] Wang M., Song Z., Liu G., Wang D. (2025). Entropic Origin of
Polymer Nucleation at Amorphous Solid Interfaces. Phys. Rev. Lett..

[19] Shen Y., Zhao Z., Li Y., Liu S., Liu F., Li Z. (2019). A facile method to prepare high molecular weight bio-renewable poly
(γ-butyrolactone) using a strong base/urea binary synergistic
catalytic system. Polym. Chem..

[20] Colonna S., Bernal M., Gavoci G., Gomez J., Novara C., Saracco G., Fina A. (2017). Effect of
processing conditions on
the thermal and electrical conductivity of poly (butylene terephthalate)
nanocomposites prepared via ring-opening polymerization. Materials & Design.

[21] Maddalena L., Benselfelt T., Gomez J., Hamedi M. M., Fina A., Wågberg L., Carosio F. (2021). Polyelectrolyte-assisted
dispersions
of reduced graphite oxide nanoplates in water and their gas-barrier
application. ACS Appl. Mater. Interfaces.

[22] Crescenzi V., Manzini G., Calzolari G., Borri C. (1972). Thermodynamics of fusion
of poly-β-propiolactone and poly-ϵ-caprolactone. comparative
analysis of the melting of aliphatic polylactone and polyester chains. Eur. Polym. J..

[23] Cohn D., Younes H., Marom G. (1987). Amorphous
and crystalline morphologies
in glycolic acid and lactic acid polymers. Polymer.

[24] Montes
de Oca H., Ward I., Chivers R., Farrar D. (2009). Structure
development during crystallization and solid-state processing of poly
(glycolic acid). J. Appl. Polym. Sci..

[25] Müller A., Michell R., Pérez R., Lorenzo A. (2015). Successive Self-nucleation
and Annealing (SSA): Correct design of thermal protocol and applications. Eur. Polym. J..

[26] Müller, A. J. ; Lorenzo, A. T. ; Arnal, M. L. Recent Advances and Applications of “Successive Self-Nucleation and Annealing” (SSA) High Speed Thermal Fractionation, Macromolecular Symposia; Wiley Online Library, 2009; pp 207–214.

[27] Müller A., Hernández Z., Arnal M., Sánchez J. (1997). Successive
self-nucleation/annealing (SSA): A novel technique to study molecular
segregation during crystallization. Polym. Bull..

[28] Keridou I., Del Valle L. J., Funk L., Turon P., Yousef I., Franco L., Puiggalí J. (2019). Isothermal crystallization kinetics
of poly (4-hydroxybutyrate) biopolymer. Materials.

[29] Hu H., Dorset D. L. (1990). Crystal structure
of poly (iε-caprolactone). Macromolecules.

[30] Chatani Y., Suehiro K., Ôkita Y., Tadokoro H., Chujo K. (1968). Structural
studies of polyesters. I. Crystal structure of polyglycolide. Die Makromolekulare Chemie: Macromolecular Chemistry and Physics.

[31] Chatani Y., Okita Y., Tadokoro H., Yamashita Y. (1970). Structural
studies of polyesters. III. Crystal structure of poly-ε-caprolactone. Polym. J..

[32] Su F., Iwata T., Sudesh K., Doi Y. (2001). Electron and X-ray
diffraction study on poly (4-hydroxybutyrate). Polymer.

[33] Su F., Iwata T., Tanaka F., Doi Y. (2003). Crystal structure and
enzymatic degradation of poly (4-hydroxybutyrate). Macromolecules.

[34] Zong X.-H., Wang Z.-G., Hsiao B. S., Chu B., Zhou J. J., Jamiolkowski D. D., Muse E., Dormier E. (1999). Structure
and Morphology
Changes in Absorbable Poly­(glycolide) and Poly­(glycolide-co-lactide)
during in Vitro Degradation. Macromolecules.

[35] Niu D., Wang H., Liu B., Xu P., Tashiro K., Yang C., Ma P. (2023). Crystal Structure of
Poly (glycolic
acid) β Form. Macromolecules.

[36] Niu D., Wang H., Ma Y., Xu P., Li J., Yang W., Liu T., Tashiro K., Lemstra P. J., Ma P. (2023). A β-Form Crystal
Modification of Poly (glycolic acid): Formation,
Stabilization, and β–α Transition. Macromolecules.

[37] Kawai A., Hamamoto N., Sasanuma Y. (2022). Conformational
characteristics and
conformation-dependent properties of poly (ε-caprolactone). Phys. Chem. Chem. Phys..

[38] Kotula A. P., Snyder C. R., Migler K. B. (2017). Determining
conformational order
and crystallinity in polycaprolactone via Raman spectroscopy. Polymer.

[39] Su F., Iwata T., Tanaka F., Doi Y. (2003). Crystal Structure and
Enzymatic Degradation of Poly­(4-hydroxybutyrate). Macromolecules.

[40] Fernandez-Tena A., Pérez-Camargo R. A., Coulembier O., Sangroniz L., Aranburu N., Guerrica-Echevarria G., Liu G., Wang D., Cavallo D., Müller A. J. (2023). Effect
of Molecular Weight on the Crystallization and Melt Memory of Poly
(ε-caprolactone)­(PCL). Macromolecules.

[41] Nakafuku C., Yoshimura H. (2004). Melting parameters of poly (glycolic acid). Polymer.

[42] Meuler A. J., Hillmyer M. A., Bates F. S. (2009). Ordered
network mesostructures in
block polymer materials. Macromolecules.

[43] Shibaev, V. P. ; Lam, L. Liquid Crystalline and Mesomorphic Polymers; Springer Science & Business Media, 2012.

[44] Allegra G., Meille S. V. (2004). Mesomorphic phases of flexible polymers: the self-compacting
chain model. Macromolecules.

