# Supplementary material for: Different Crystalline Populations for Biopolyesters within Graphene-Based Nanopapers
Source: Macromolecules. 2026 Mar 2;59(5):2780–92. doi: 10.1021/acs.macromol.5c03526 (PMC12981308; doi:10.1021/acs.macromol.5c03526)
Supplement: Supplementary file 1 [file ma5c03526_si_001.pdf]

Supporting information

Different Crystalline Populations for biopolyesters within Graphene-Based Nanopapers

Hui Zhao[1], Ricardo A. Pérez-Camargo[2], Yongzheng Li[3], Zhibo Li[3], Guoming Liu[4,5]*, Alejandro J. Müller[2,6]* and Alberto Fina*[1]

[1] Dipartimento di Scienza Applicata e Tecnologia, Politecnico di Torino- Alessandria campus, viale Teresa Michel, 5, 15121 Alessandria, Italy

[2] POLYMAT and Department of Polymers and Advanced Materials: Physics, Chemistry and Technology, Faculty of Chemistry, University of the Basque Country UPV/EHU, Paseo Manuel de Lardizabal 3, 20018, Donostia-San Sebastián, Spain

[3] State Key Laboratory of Advanced Optical Polymer and Manufacturing Technology; College of Polymer Science and Engineering, Qingdao University of Science and Technology, 266042, Qingdao, China

[4] University of Chinese Academy of Sciences, Beijing 100049, China

[5] Beijing National Laboratory for Molecular Sciences, Institute of Chemistry, Chinese Academy of Sciences, Beijing 100190, China

[6] IKERBASQUE, Basque Foundation for Science, Bilbao, 48009, Spain

*Corresponding authors: alberto.fina@polito.it; alejandro.j.mueller@gmail.com; gmliu@iccas.ac.cn

1. Calculation of 100% Crystallization Enthalpy of P4HB by Group Contribution Method

The CO group has a contribution of 0 kJ/mol.

The -CH_2- group contributes 4 kJ/mol.

The -O- group has a contribution of 1 kJ/mol

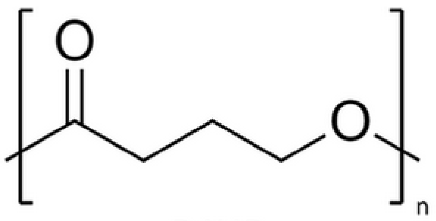

P4HB

The molecular weight of the repetitive unit is: 4 Cx12.01 g/mol + 2Ox16gmol + 6Hx1.01g.mol = 86.1 g/mol.

Next, employing equation 1:

$$\Delta H_m^0 = \frac{\Delta H_{CH2}.n_{CH2} + \Delta H_O + \Delta H_{CO}}{M_i} \qquad (1)$$

$$\Delta H_m^0 = \frac{\frac{3x4kJ}{mol} + \frac{1x1kJ}{mol} + 0kJ/mol}{86.1\ g/mol} = \frac{13000J/mol}{86.1g/mol} = 151\frac{J}{g} \qquad (2)$$

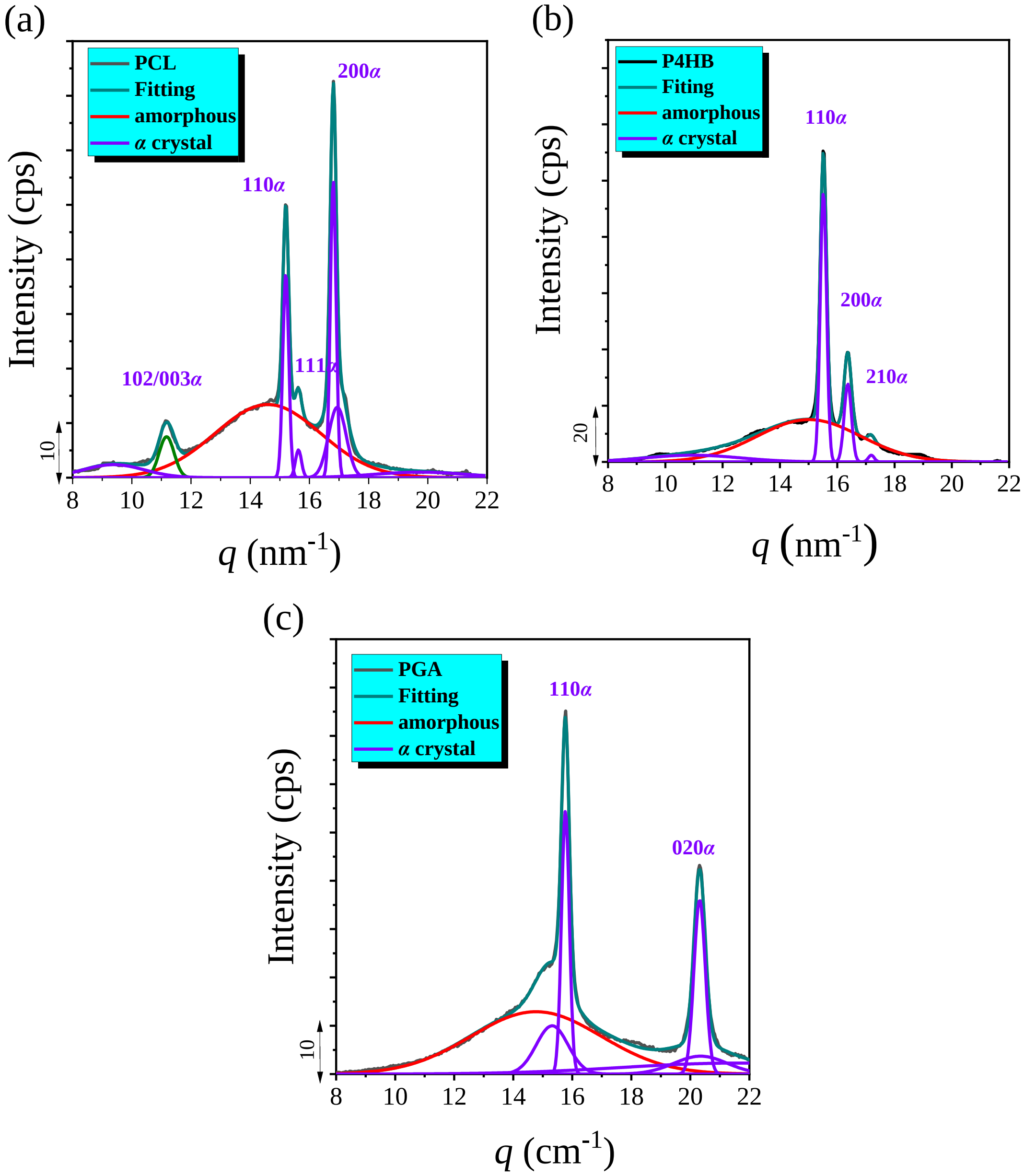

Figure S1. Example illustrating the fitting of the WAXS profile of (a) PCL, (b) P4HB and (c) PGA.

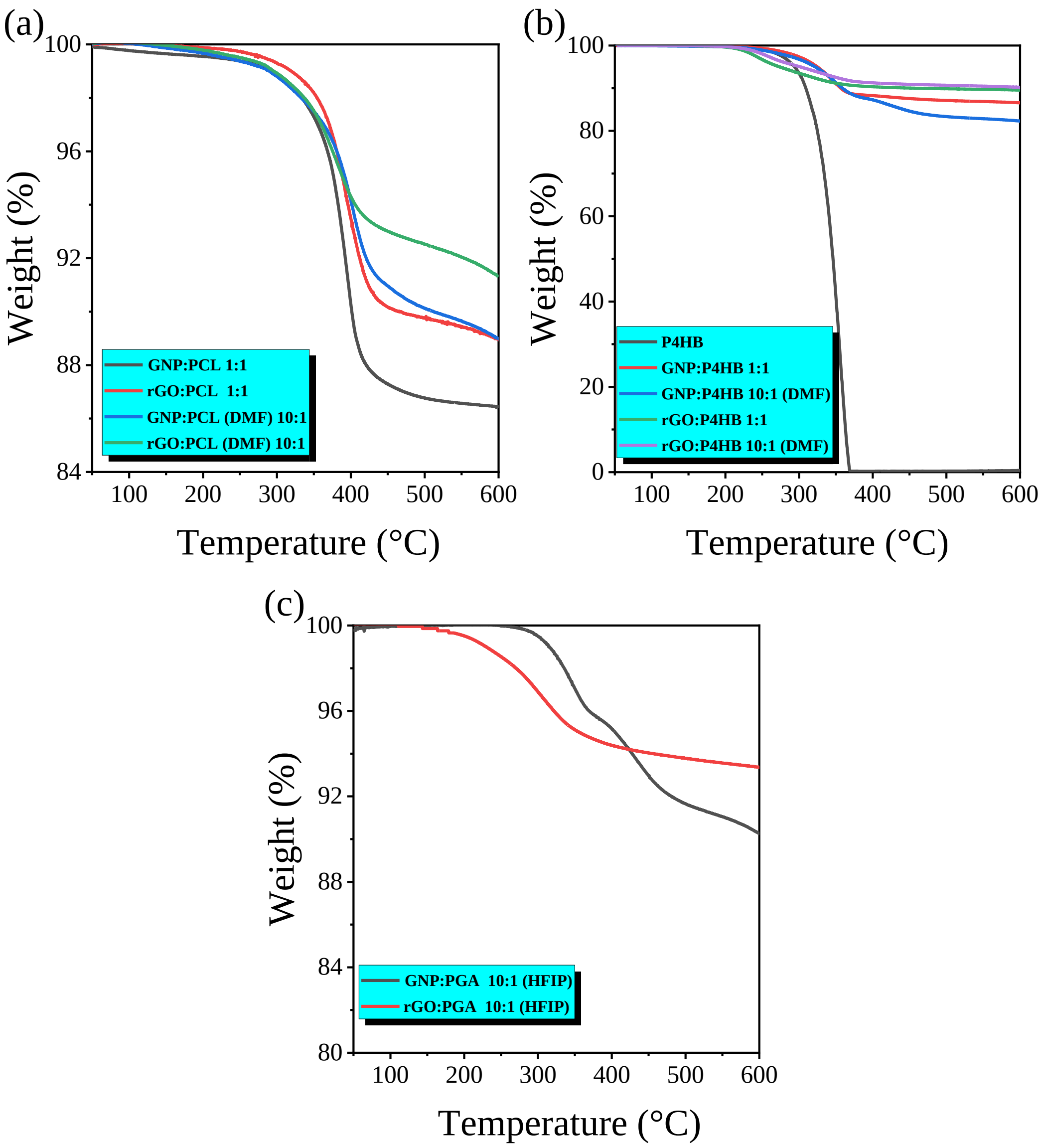

Figure S2. Thermogravimetric analysis curves of polymer nanopapers prepared with different methods. (a) GRM-PCL, (b) GRM-P4HB and (c) GRM-PGA.

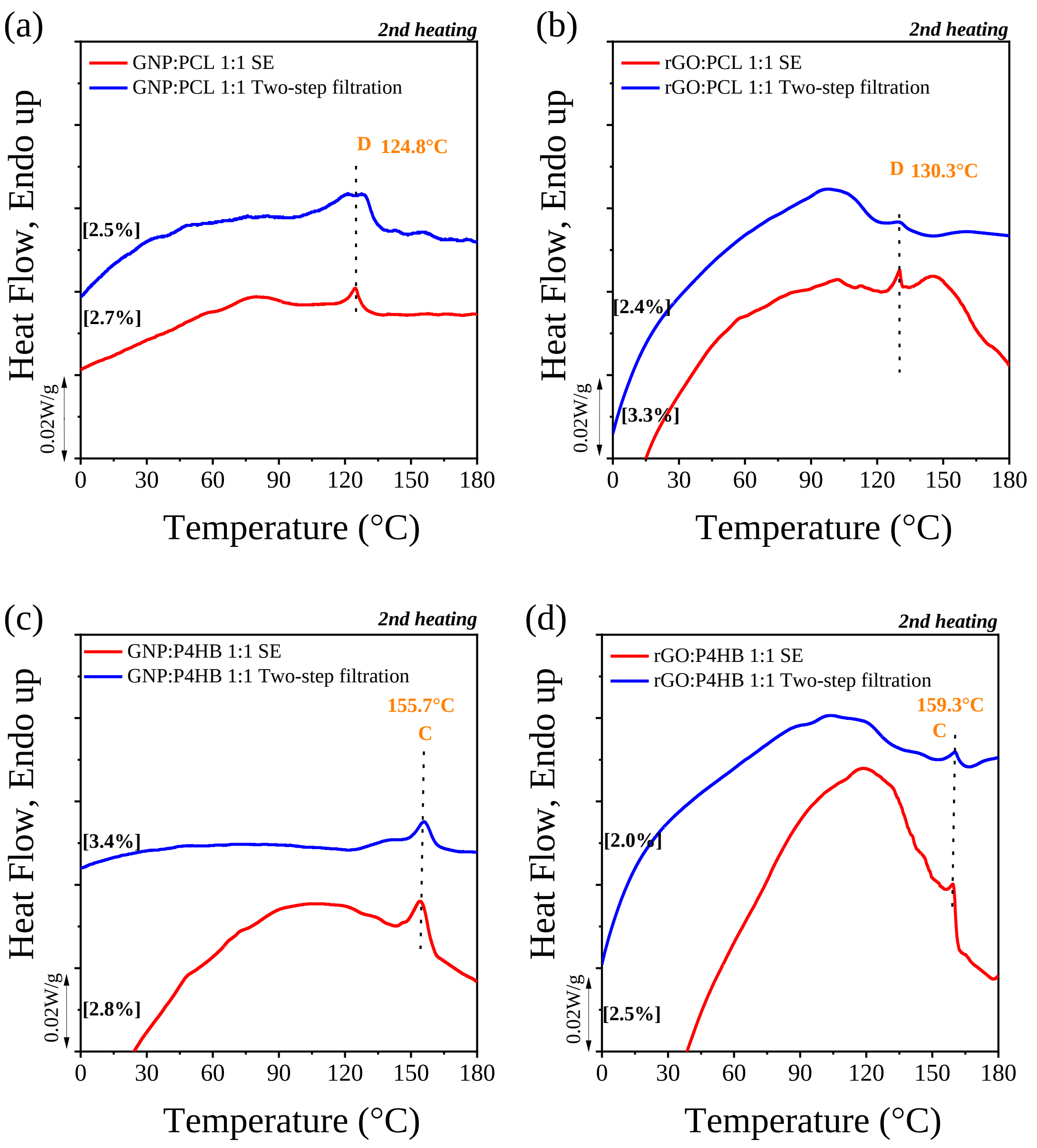

Figure S3. The thermodynamic behavior of nanopaper is prepared by filtration method and two-step filtration method. (a) GNP-PCL and (b) rGO-PCL, (c) GNP-P4HB and rGO-P4HB. In brackets are the polymer contents from TGA (reported in Figures S4, S5).

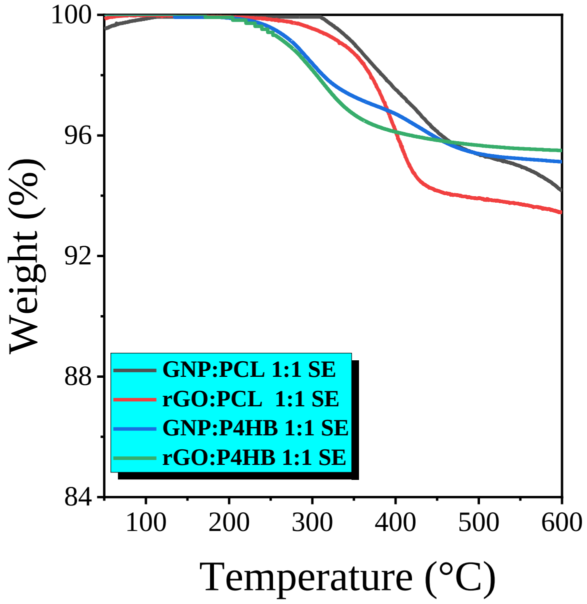

Figure S4. Thermogravimetric analysis curves of the thermal weight loss (TGA) with different GRM nanopapers from Soxhlet extraction later.

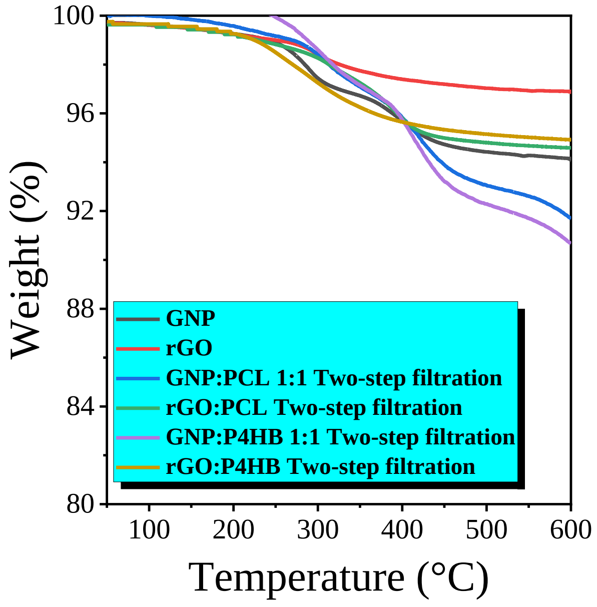

Figure S5. Thermogravimetric analysis curves of the thermal weight loss (TGA) thought the nanopaper preparation method of two-step filtration.

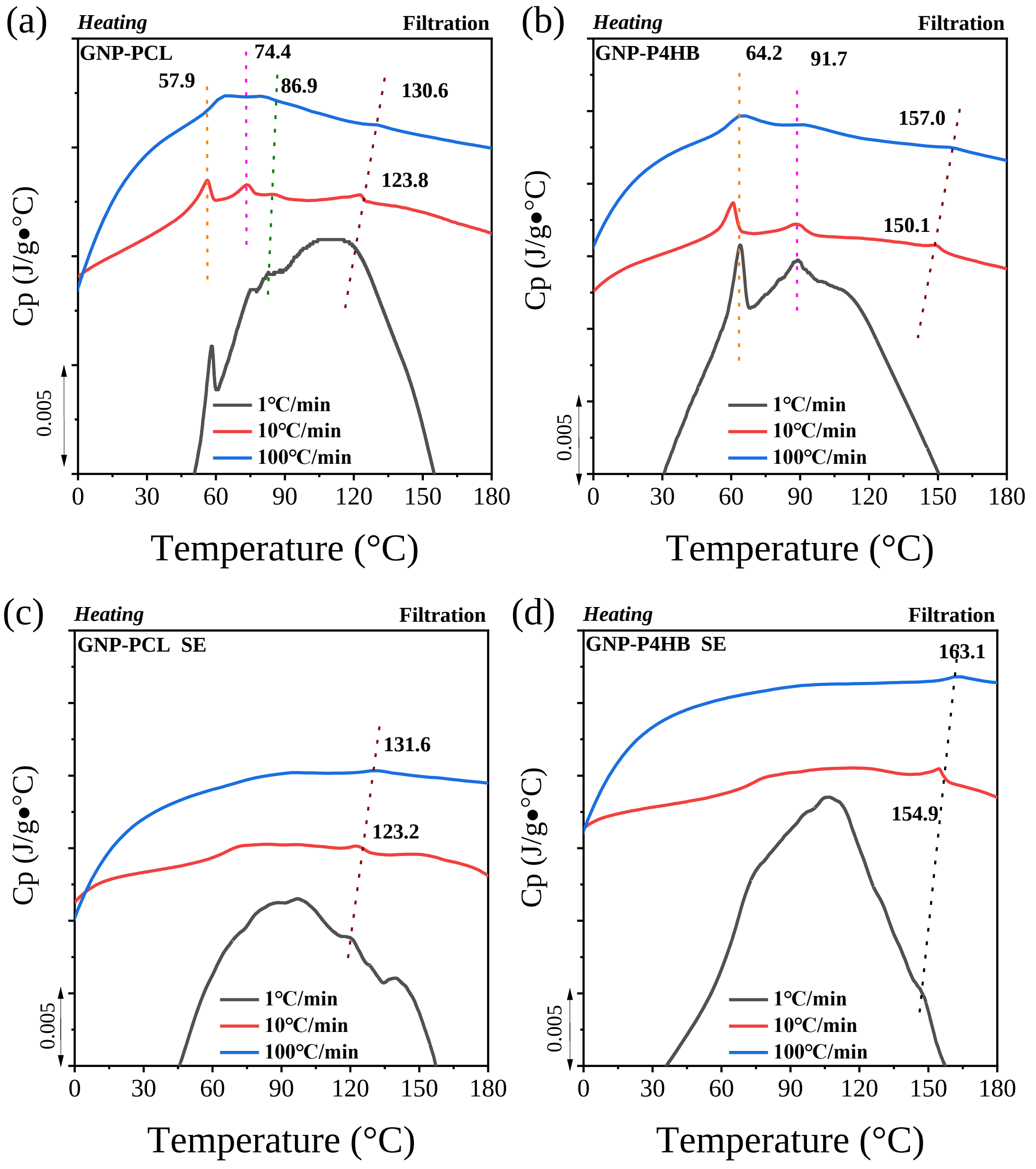

Figure S6. The DSC curves of nanopapers with different heating rate (1°C/min, 10°C/min and 100°C/min), (a) GNP-PCL, (b) GNP-P4HB, (c) GNP-PCL SE and (d) GNP-P4HB SE.

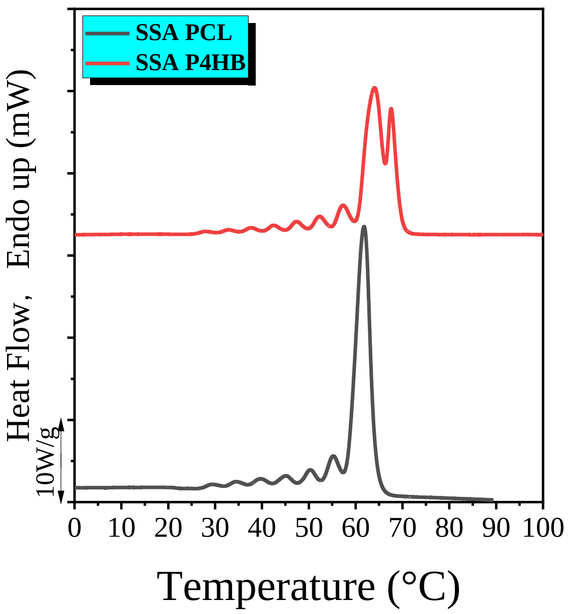

Figure S7. Final heating curves of SSA of PCL and P4HB.

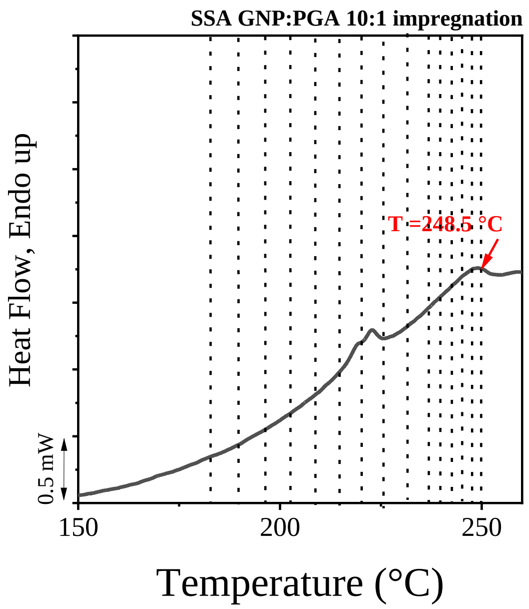

Figure S8. SSA final heating for PGA/GNP nanopaper by impregnation method.

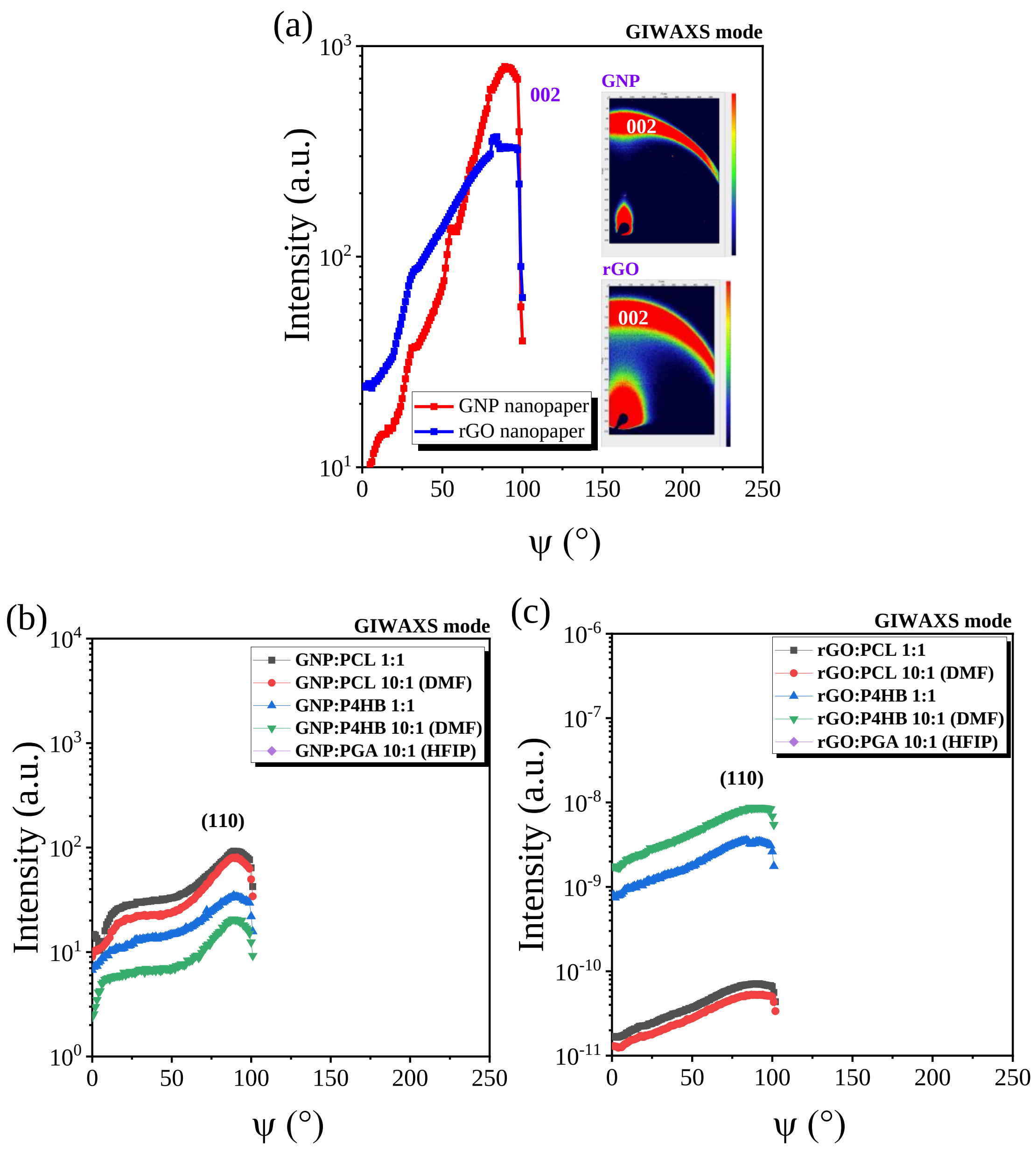

Figure S9. (a) Intensity distribution of selected reflections vs. azimuthal angle for GNP and rGO nanopapers obtained from the reflections of the out-of-planes (002). Intensity distribution of selected reflections vs. azimuthal angle for a series of nanopapers with different polymers obtained from the reflections of the out of -planes (110). (b) GNP and (b) rGO.

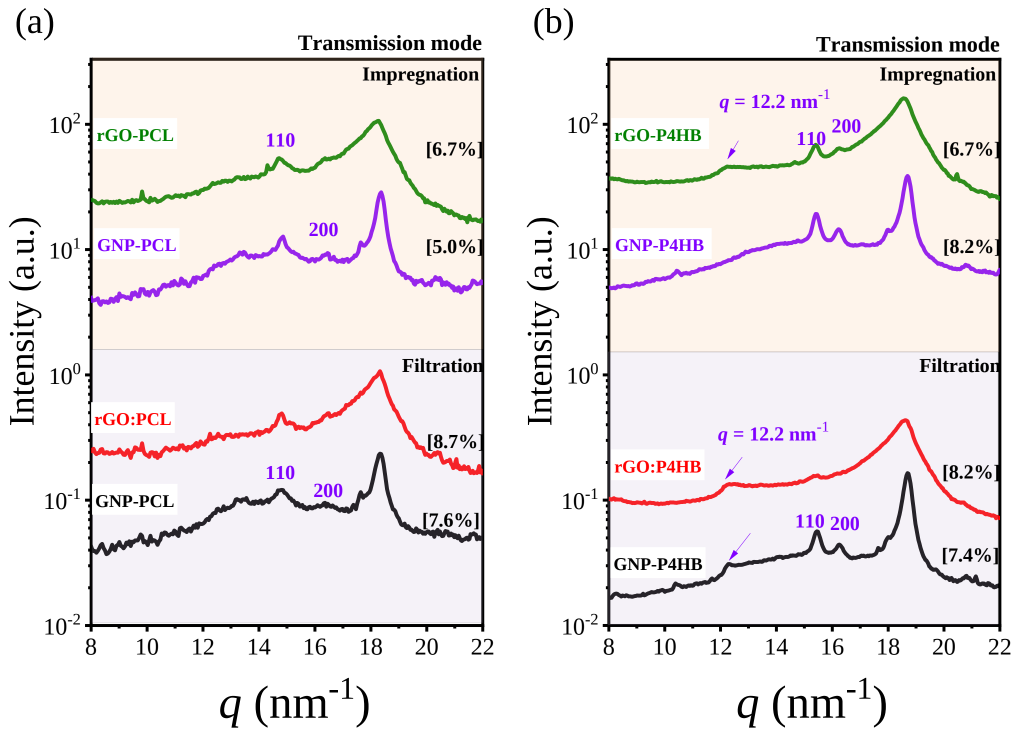

Figure S10. WAXS curves of GRM-based PCL and P4HB nanopapers in transmission mode. (a) GRM/PCL and (b) GRM/P4HB. An obvious difference is obtained for the (002) planes in GRM at 18.4 nm^{-1}, in which the peak is much broader in rGO as compared to GNP. This confirms the higher structural disorder in rGO. Two peaks are observed, corresponding to the (110) and (200) reflections of PCL *α* crystals and P4HB *α* crystals.

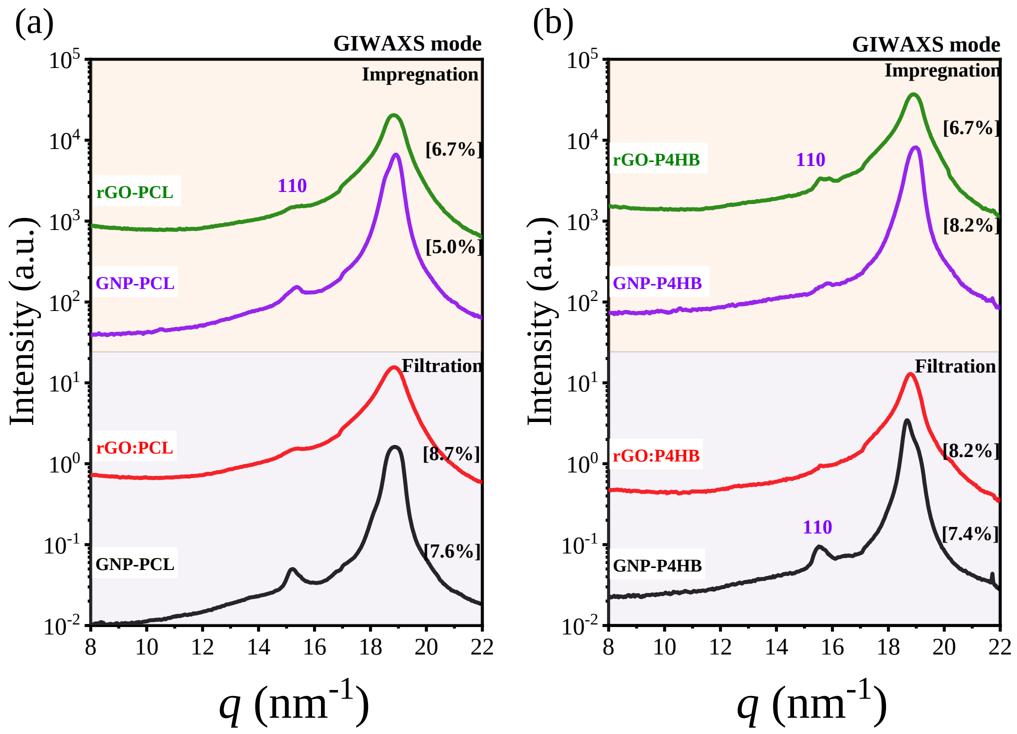

Figure S11. WAXS curves of GRM-based PCL and P4HB nanopapers in GIWAXS mode. (a) GRM/PCL and (b) GRM/P4HB. The 110-polymer signal in the samples prepared by the impregnation method is lower compared to transmission, while the crystal orientation in rGO nanopaper is even weaker.

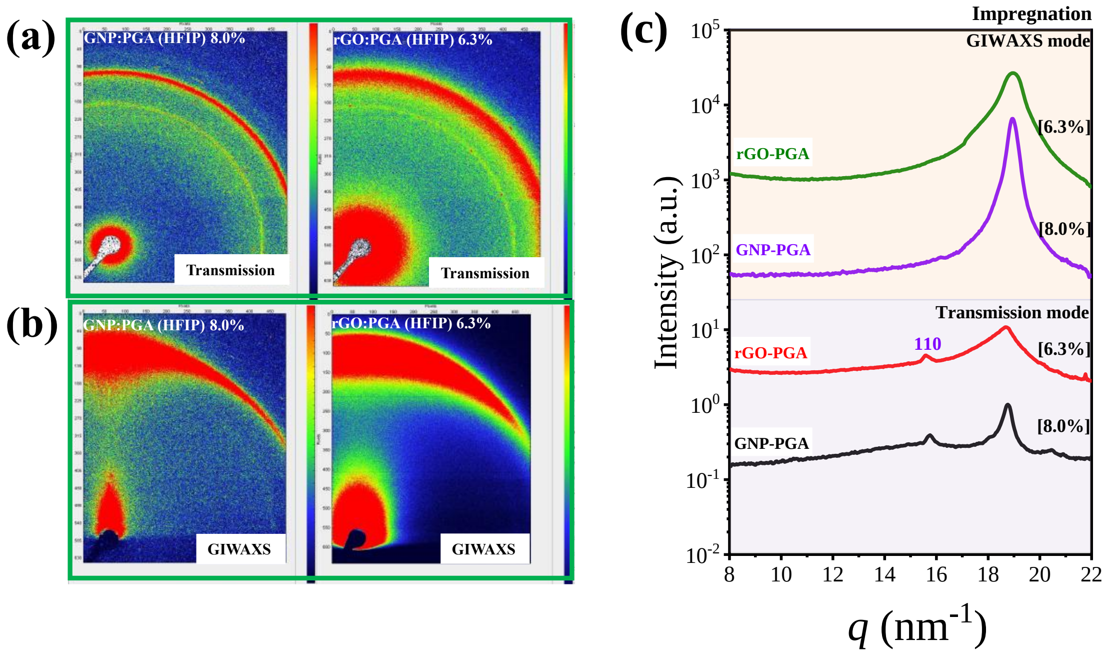

Figure S12. 2D WAXS images of PGA nanopaper prepared by impregnation method with different GRMs in different directions. (a) Transmission mode and (b) GIWAXS mode. (c) Transmission and GIWAXS plots for GRM-PGA prepared by impregnation. In brackets are the polymer contents.

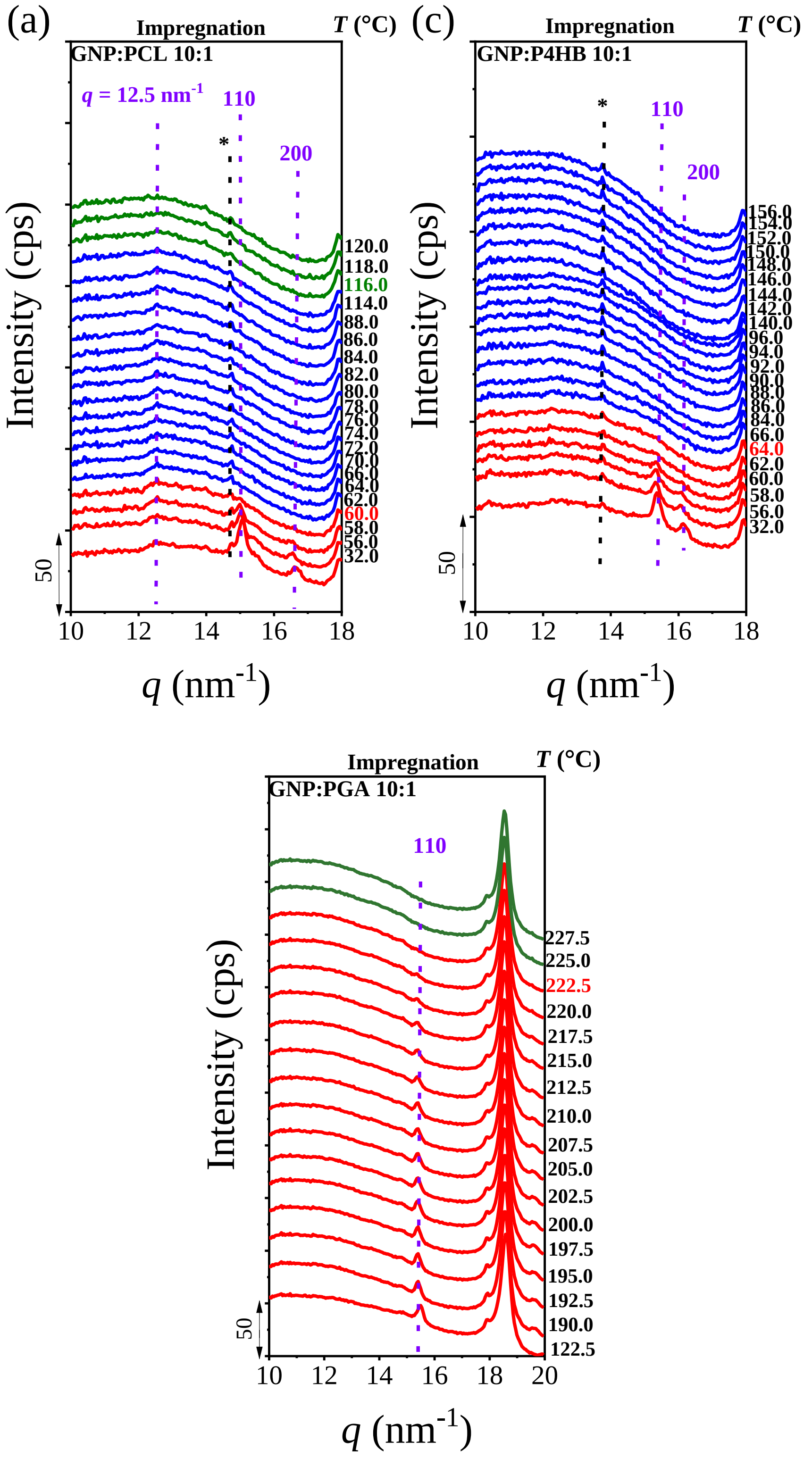

Figure S13. Variable temperature WAXS curves of PCL, P4HB, PGA nanopaper prepared by impregnation method, (a) GNP-PCL, (b) GNP-P4HB and (c) GNP-PGA.

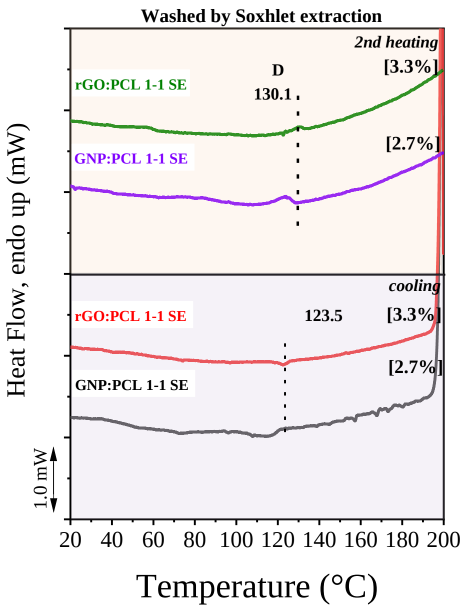

Figure S14. DSC curves of PCL nanopapers on GNP or rGO after Soxhlet extraction. The black square in the Figure indicates the polymer content from TGA in Figure S4.
